# Shared and distinct structural brain networks related to childhood maltreatment and social support: connectome-based predictive modeling

**DOI:** 10.1038/s41380-023-02252-3

**Published:** 2023-09-15

**Authors:** Alexandra Winter, Marius Gruber, Katharina Thiel, Kira Flinkenflügel, Susanne Meinert, Janik Goltermann, Nils R. Winter, Tiana Borgers, Frederike Stein, Andreas Jansen, Katharina Brosch, Adrian Wroblewski, Florian Thomas-Odenthal, Paula Usemann, Benjamin Straube, Nina Alexander, Hamidreza Jamalabadi, Igor Nenadić, Linda M. Bonnekoh, Katharina Dohm, Elisabeth J. Leehr, Nils Opel, Dominik Grotegerd, Tim Hahn, Martijn P. van den Heuvel, Tilo Kircher, Jonathan Repple, Udo Dannlowski

**Affiliations:** 1 Institute for Translational Psychiatry, University of Münster, Münster, Germany; 2 Department of Psychiatry, Psychosomatic Medicine and Psychotherapy, University Hospital Frankfurt, Goethe University, Frankfurt, Germany; 3 Institute for Translational Neuroscience, University of Münster, Münster, Germany; 4 Department of Psychiatry and Psychotherapy, University of Marburg, Marburg, Germany; 5 Center for Mind, Brain and Behavior (CMBB), University of Marburg and Justus Liebig University Giessen, Giessen, Germany; 6 Core-Facility Brainimaging, Faculty of Medicine, University of Marburg, Marburg, Germany; 7 Department of Child and Adolescent Psychiatry, University Hospital Münster, Münster, Germany; 8 Department of Psychiatry and Psychotherapy, University of Jena, Jena, Germany; 9 Connectome Lab, Department of Complex Trait Genetics, Center for Neurogenomics and Cognitive Research, Vrije Universiteit Amsterdam, Amsterdam Neuroscience, Amsterdam, The Netherlands; 10 Department of Child Psychiatry, Amsterdam University Medical Center, Amsterdam Neuroscience, Amsterdam, The Netherlands

**Keywords:** Neuroscience, Molecular biology

## Abstract

Childhood maltreatment (CM) has been associated with changes in structural brain connectivity even in the absence of mental illness. Social support, an important protective factor in the presence of childhood maltreatment, has been positively linked to white matter integrity. However, the shared effects of current social support and CM and their association with structural connectivity remain to be investigated. They might shed new light on the neurobiological basis of the protective mechanism of social support. Using connectome-based predictive modeling (CPM), we analyzed structural connectomes of *N* = 904 healthy adults derived from diffusion-weighted imaging. CPM predicts phenotypes from structural connectivity through a cross-validation scheme. Distinct and shared networks of white matter tracts predicting childhood trauma questionnaire scores and the social support questionnaire were identified. Additional analyses were applied to assess the stability of the results. CM and social support were predicted significantly from structural connectome data (all *r*s ≥ 0.119, all *ps* ≤ 0.016). Edges predicting CM and social support were inversely correlated, i.e., positively correlated with CM and negatively with social support, and vice versa, with a focus on frontal and temporal regions including the insula and superior temporal lobe. CPM reveals the predictive value of the structural connectome for CM and current social support. Both constructs are inversely associated with connectivity strength in several brain tracts. While this underlines the interconnectedness of these experiences, it suggests social support acts as a protective factor following adverse childhood experiences, compensating for brain network alterations. Future longitudinal studies should focus on putative moderating mechanisms buffering these adverse experiences.

## Introduction

Early aversive experiences, such as childhood maltreatment, increase the likelihood of impaired behavioral, cognitive, emotional, and social development with long-term consequences lasting into adulthood [1, 2]. Physical and emotional neglect as well as sexual, physical, and emotional exploitation are even manifested on a neurobiological level as they have been linked to altered brain structure and function [3–7]. Following network-based theories on brain organization [8–10], microstructural and graph theory-based measures of white matter structural connectivity have gained substantial importance in the last few years. Studies analyzing the human structural connectome, i.e., the network of all brain regions, rely on diffusion tensor imaging (DTI) which investigates white matter fibers connecting gray matter regions. They follow the assumption that healthy psychosocial human functioning arises from healthy networks of interacting brain regions [9]. Consequently, a risk factor such as maltreatment might compromise psychosocial functioning by targeting such brain networks, rendering network-level brain analyses mandatory for identifying maltreatment-related alterations of brain structural network communication.

Only a few studies have investigated structural connectome alterations as a consequence of childhood maltreatment, providing preliminary evidence that adverse early experiences alter communication of brain areas [11]. Maltreatment-related disruptions of these connections are detectable as early as in childhood [12]. This aspect becomes even more important considering that childhood and adolescence are particularly vulnerable phases for the formation of the connectome [13]. Studies on white matter microstructure revealed reduced white matter integrity in maltreated vs. non-maltreated adolescents [14]. Results from older individuals tentatively suggest that these changes last into adulthood, and occur irrespective of the presence of a psychiatric diagnosis (for a meta-analysis see refs. [15–20].

Ohashi and colleagues [21] made a first attempt to identify compensatory brain alterations which might enable individuals with a history of childhood maltreatment to successfully ward off psychopathology. Asymptomatic, or “resilient”, individuals showed no significant difference in global network architecture as compared to “susceptible”, symptomatic childhood maltreatment-exposed participants. However, they exhibited significantly lower nodal efficiency of the right amygdala, that is, a diminished ability to spread information throughout adjacent networks.

Therefore, individual protective factors likely play a role in countering the neural effects of harmful environmental influences. One source of resilience can be the perception of a current helpful social environment: social support is crucial for good psychological health in the presence of genetic, developmental, but also environmental risks [22–24], such as childhood maltreatment [25–27]. Despite this importance, the effects of social health on structural connectivity have been investigated less extensively, as compared to childhood maltreatment. A few studies, even though heterogeneous in methodology and applied constructs, cautiously imply a positive association between social health and white matter integrity in healthy adults [28–30]), e.g., between fronto-temporal white matter tracts and the size of one’s social network [31]. One study revealed a positive association of perceived social support in older adults with more intact white matter microstructure [32]. To the best of our knowledge, no studies have been published investigating shared structural connectome signatures of social support and childhood maltreatment.

In summary, regarding research on structural connectome alterations, there are only preliminary findings showing effects of childhood maltreatment, and a lack of studies investigating the shared and distinct effects of social support and childhood maltreatment on the structural connectome. Therefore, we aimed to examine the association between childhood maltreatment, social support and their overlap on brain structural connectome measures using connectome-based predictive modeling (CPM). This recent but validated method employs predictive models of brain-behavior relationships from connectivity data by selecting the most relevant connections, i.e., edges, across the whole brain. The main advantages comprise cross-validation (CV) including a training and a novel test data set. A natural data structure with continuous variables and linear operations in a whole-brain approach can be used. Lastly, the results are easily interpreted, as the correlation between true and predicted values is evaluated as an indicator of a good predictive model [33]. While standard analyses, such as the network-based statistic toolbox [34], provide group-level inference on the association between connectome and a behavioral phenotype, CPM uses CV to provide information on the utility of the (structural) connectome for a case-by-case prediction of behavioral phenotypes in unseen data. CPM has been used previously to predict phenotypes from a broad range of domains, e.g., states like loneliness [35], diagnostic traits like anxiety [36, 37], but also protective factors like cognitive reserve [38]. One study used CPM to predict childhood maltreatment scores based on resting-state functional connectivity [39]. Nonetheless, the more general aspect of social health, let alone particularly social support, has not been investigated with CPM yet.

Following up and expanding upon previous literature, we hypothesized a significant association of childhood maltreatment and measures of structural connectivity in a large healthy adult sample using a continuous variable approach. Moreover, we also expected a significant effect of social support on brain networks. Since we also intended to investigate how protective social support and early-life adversities interact on a neural network-level, we exploratively tested for a shared connectome signature of childhood maltreatment and social support.

## Methods and material

### Participants

The sample was drawn from the Marburg-Münster Affective Disorders Cohort Study (MACS), recruited as part of the bi-centric FOR2107 study across the universities of Marburg and Münster, Germany. Participants were invited with flyers and newspaper advertisements in the vicinity of Marburg and Münster, Germany. For the present study, participants with a history of a mental disorder, verbal IQ < 80, history of head trauma or unconsciousness, current intake of psychopharmacological medication, and severe neurological illness were excluded. All participants from the MACS, who did not meet these exclusion criteria and provided all necessary data were included in the present analysis to ensure maximal statistical power, resulting in a final sample of *N* = 904 healthy participants (Supplement 1).

Participants gave written informed consent to the study protocol before participation and received financial compensation. The FOR2107 study was approved by the Ethics Committees of the Medical Faculties, University of Marburg (AZ: 07/14) and University of Münster (AZ: 2014–422-b-S).

### Materials

Participants underwent the Structured Clinical Interview (SCID-I) to ensure the absence of any lifetime Axis I Disorder (DSM-IV-TR) [40] (for a detailed overview and across-site harmonization efforts see ref. [41]).

The German version of the 28-item Childhood Trauma Questionnaire (CTQ; [42]) collected participants’ retrospective self-reported adverse childhood experiences. It consists of five subscales with five items each (physical abuse, physical neglect, emotional abuse, emotional neglect, and sexual abuse) which contribute to the sum score. Individual scores have proven to be highly stable even over a two-year span [43]. The internal consistency in our sample was high (Cronbach’s α = 0.90 (25 Items)). See Table 1 for an overview of demographics and questionnaire scores.

**Table 1:** Sociodemographic characteristics of the sample.

Characteristic	Healthy adults (*N* = 904)	
	*N* or *M* (*SD*)	Min-Max
Sex, m/f	322/582	–
Marburg/Münster	530/374	–
Age	34.04 (12.84)	18–65
Childhood trauma questionnaire (CTQ)		
Sum score	32.60 (8.73)	25–90
Physical abuse	5.59 (1.52)	5–18
Emotional abuse	7.05 (2.87)	5–24
Sexual abuse	5.28 (1.44)	5–20
Physical neglect	6.19 (1.89)	5–18
Emotional neglect	8.50 (3.71)	5–25
Perceived social support (FSozU)	4.51 (0.54)	1.55–5.00
Social functioning (SF-36)^a^	91.64 (14.93)	12.5–100.0
Total intracranial volume (TIV)	1526.51 (144.61)	1127.62–2120.19

Numbers represent the total number of participants, or Mean ± Standard Deviation.

*FSozU* social support questionnaire (“Fragebogen zur sozialen Unterstützung”), *SF-36* short form 36 health survey.

^a^Data available for 902 participants.

To estimate perceived social support, the German questionnaire of social support (“Fragebogen zur sozialen Unterstützung”; FSozU; [44]) was administered (Supplement 1). This 22-item questionnaire captures the subjective experience of receiving support from the social network, or the ability to draw on resources from the social environment, e.g., family, friends, but also colleagues and neighbors. The instructions do not refer to any specific past period of time but rather enquire about the current perception. The internal consistency was proven to be high in a representative sample (Cronbach’s α = 0.91; [44]) and equally high in the present sample (Cronbach’s α = 0.93). Individuals from the current sample participated from 2014 to 2018, therefore, measures of social support were not influenced by COVID-19 pandemic-related social restrictions. The 2-item subscale “social functioning” of the short form 36 health survey (SF-36) was additionally used to obtain limitations in social activities due to physical or emotional problems in the last four weeks, i.e., a measure of current social functionality in everyday life [45].

### MRI data acquisition and preprocessing

Participants were examined on a 3T whole body MRI scanner (Marburg: Tim Trio, Siemens, Erlangen, Germany; Münster: Prisma fit, Siemens, Erlangen, Germany; see Supplement 2 for acquisition parameters and preprocessing of diffusion-weighted images, and details on the reconstruction of the anatomical connectome). Briefly, we reconstructed the edges (defined as the number of white matter streamlines) connecting 114 nodes (defined as cortical brain regions from the Cammoun subdivision of the Desikan-Killiany atlas [46, 47]) using CATO [48], resulting in a connectome of 114*113/2 = 6441 edges. Each participant’s connectome was stored in a connectivity matrix (for quality control see Supplement 3), with rows and columns representing nodes, and matrix entries representing edges. Edges were considered if they comprised at least three reconstructed streamlines to balance the sensitivity and specificity of the resulting connectivity matrices [49, 50]. Edges that connected the same node at both ends were excluded from our connectivity matrices.

### Statistical analyses

Demographic data were analyzed with SPSS 27. For CPM analysis, Matlab 2019b was used. Age, sex, total intracranial volume (TIV), and head motion were covariates of no interest in all analyses. Head motion was retrieved from FSL’s eddy_movement_rms output by averaging the volume-specific displacement (defined as the square root of the average squared displacement of each voxel within a given volume relative to the previous volume) across all volumes from a given participant.

### Connectome-based predictive modeling

**Fig. 1:**
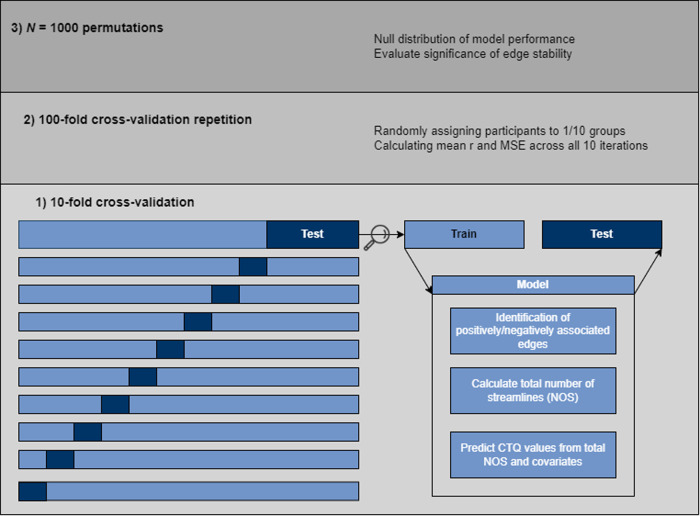
Schematic illustration of the CPM analysis procedure. Note. Ten-fold cross-validation was repeated 100 times, followed by permutation testing.

We employed two CPM analyses to separately predict values from the CTQ and FSozU from structural connectivity matrices. We will briefly describe the CPM analysis conducted to predict the CTQ values (Fig. 1). We applied the same procedure to predict FSozU values. Following the CPM approach established by Shen et al. [33], we used a ten-fold CV scheme for the CPM analysis. To this end, data were divided into ten groups. In each of the ten iterations of the CV scheme, data from nine groups were used as the training set, and data from the remaining group were used as the test set. An iteration of the CV scheme proceeded as follows: First, following previous connectome studies (e.g. [51]), we applied one-tailed one sample *t*-tests to each edge and included only those edges that deviated significantly (*p* < 0.05) from zero across all participants. This step was included to retain only highly consistent edges and was done within each CV iteration to avoid data leakage. Second, utilizing Spearman partial correlations, we identified edges that were either positively or negatively associated with the CTQ in the training set (*p* < 0.05), while correcting for the above-mentioned covariates. Third, we separately calculated the total number of streamlines (NOS) across positive and negative edges. Fourth, two linear models were fitted in the training set to predict the CTQ values from the total NOS in positive and negative edges, respectively, while correcting for the above covariates. Fifth, the trained models were used to predict the CTQ values in the test set. Sixth, performance of the predictive model was evaluated by calculating the correlation and the mean absolute error (MAE) for the true and predicted CTQ values of the test set.

Extending the method of Shen et al. (31), 100 repetitions of a tenfold CV were implemented, resulting in a repeated CV scheme, following established guidelines [51–54]. Each of these repetitions included 1) randomly reassigning the participants to one of the ten groups used for CV and 2) calculating the average model performance across all ten CV iterations. This allowed us to establish the stability of the model performance by calculating the mean correlation and MAE across all CV iterations together with corresponding confidence intervals. Additionally, we estimated edge stability as the proportion of all 10 * 100 = 1000 CV iterations in which an edge was included in the predictive model. This value ranged from 0 to 1, with 1 indicating model inclusion of the respective edge across all CV iterations and, hence, high importance of the respective edge for predicting the target variable.

Non-parametric permutation tests were employed to assess the significance of the model performance [33]. To this end, the above CV scheme was repeated 1000 times while randomly shuffling the CTQ values across the subjects to obtain a null distribution of model performance. The *p*-value was calculated as the proportion of permutations in which the average performance of the permuted model exceeded the average performance of the predictive model. In addition, we used the permutation tests to evaluate the significance of the edge stability by calculating the proportion of permutations in which a given edge had a higher stability in the permuted model than in the predictive model.

### Exploratory analysis of network overlap

In an exploratory analysis, we evaluated the overlap of the structural brain networks associated with CTQ and FSozU. To this end, we first counted the number of edges that 1) were included in the predictive models for both CTQ and FSozU and 2) had a significant (uncorrected *p* < 0.05) edge stability. To evaluate the significance of the overlap between the two networks, we implemented a permutation test. More specifically, we drew two random sets of edges matching the number of edges of the two identified networks, and calculated the overlap between the two random sets. This procedure was repeated 1000 times to obtain a null distribution of overlaps of random edge sets. The *p*-value of the observed overlap was then calculated as the number of permutations in which the overlap between the random edge sets was higher than the observed overlap between our identified networks, divided by the total number of permutations, i.e., 1000. We additionally tested the reciprocal relationships of FSozU and CTQ, i.e., if the CTQ sum score was associated with FSozU networks, and vice versa (Supplement 4). Due to the significant shared variance of the CTQ and FSozU (*r*(902) = −0.370, *p* < 0.001; Supplement 5), we conducted a confirmatory factor analysis to verify the independence of the two constructs, CM and social support (Supplement 6).

## Results

**Fig. 2:**
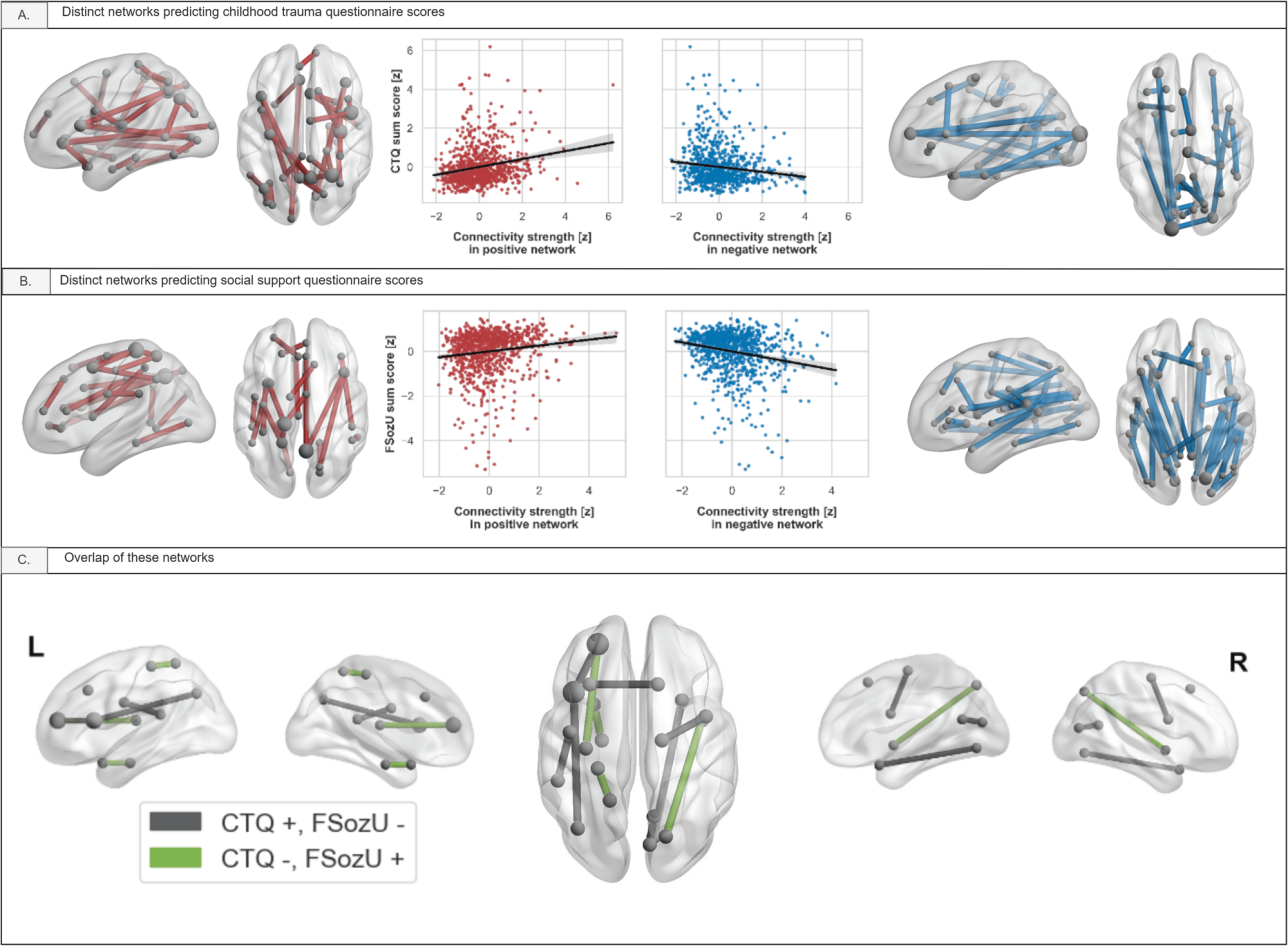
Networks distinctly associated with CTQ, FSozU, and their overlap. Note. Networks associated with childhood maltreatment (**A**) and social support (**B**) only, respectively, and scatterplots of the respective associations. Networks which are positively associated with the respective variable appear red, networks which are negatively associated blue. **C** shows the significant overlap of these networks; edges colored black represent positive association with childhood maltreatment and negative with social support, and vice versa for edges colorized green.

CPM analysis revealed a positive and a negative network, i.e., in which edge-wise connectivity strength was respectively positively or negatively correlated with CTQ, that significantly predicted CTQ values of the participants (positive: *r* = 0.224, 95%-CI [0.215; 0.233], *p* < 0.001, MAE = 6.027, 95%-CI [5.974; 6.081], *p* < 0.001; negative: *r* = 0.233, 95%-CI [0.223; 0.243], *p* < 0.001, MAE = 5.968, 95%-CI [5.911; 6.025], *p* < 0.001). Stability analyses revealed that 64 edges (corresponding to 0.99% of the entire connectome) had a significant (i.e., higher than would be expected for random data) stability across the CV iterations. Edges showing a negative correlation and significant stability connected mainly frontal and parietal brain regions, while edges with a positive correlation and significant stability connected predominantly frontal, temporal, and parietal regions, indicating a more widespread effect in the positive compared to the negative CTQ network (Fig. 2; Supplement 7).

For social support, CPM analyses revealed a positive and a negative network, i.e., in which edge-wise connectivity strength was either positively or negatively correlated with the FSozU, which significantly predicted FSozU values of the participants (positive: *r* = 0.149, 95%-CI [0.141; 0.157], *p* < 0.001, MAE = 0.386, 95%-CI [0.383; 0.389], *p* < 0.001; negative: *r* = 0.119, 95%-CI [0.109; 0.128], *p* = 0.002, MAE = 0.392, 95%-CI [0.389; 0.395], *p* = 0.036). Stability analyses revealed that 68 edges (1.06% of the connectome) had a significant stability in predicting FSozU (Supplement 8). Edges showing a positive correlation with FSozU and significant stability connected mainly parietal and frontal brain regions. Edges showing a negative correlation and significant stability connected mainly parietal and temporal brain regions (Fig. 2; Supplement 8).

While connectivity strength within the CTQ networks was correlated to FSozU and connectivity strength within the FSozU networks was correlated to CTQ, these correlations faded when controlling for CTQ and FsozU, respectively (Supplement 4), indicating predictive value of the networks specifically for the respective measure.

### Explorative analysis of network overlap

**Fig. 3:**
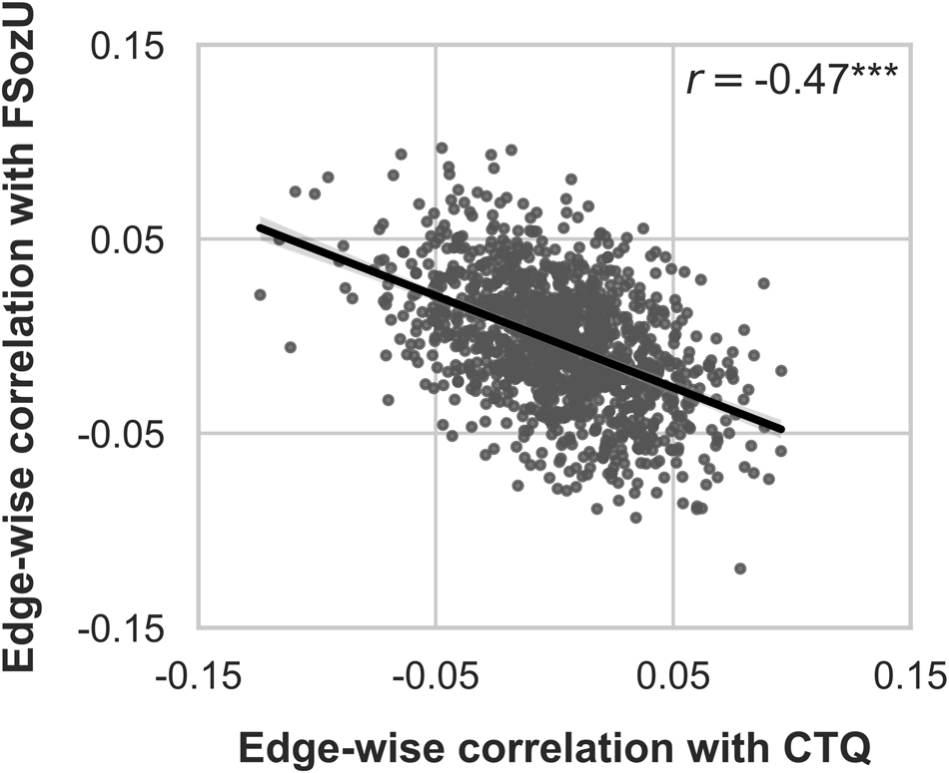
Edge-wise correlations with childhood maltreatment and social support. Note. Figure 3 depicts the correlation of edge-wise correlations with childhood maltreatment (CTQ) and social support (FSozU).

When viewed from a connectome-wide perspective, i.e., considering all edges included in our analyses irrespective of edge stability there was an inverse relationship between edge-wise correlations with CTQ and FSozU, meaning that edges positively correlated with CTQ were negatively correlated with FSozU and vice versa (*r* = −0.470, *p* < 0.001, Fig. 3).

Focusing on edges with a significant (i.e., higher than would be expected for random data) stability across the CV iterations revealed that overlapping edges were again exclusively inversely correlated. To be precise, 11 edges with significant edge stability were predictive of CTQ and FSozU, with seven of them being positively correlated with CTQ and negatively correlated with FSozU, and four of them being negatively correlated with CTQ and positively correlated with FSozU (Fig. 2; Supplement 9).

### Robustness checks

Given the skewness of both CTQ and FSozU (for scatter plots see Supplements 7 and 8), we conducted additional analyses excluding multivariate outliers with no significant change in results (Supplement 10). Furthermore, we repeated the analyses with quadratic age and global connectivity strength (NOS) instead of TIV as covariates, respectively, yielding the same results (Supplements 11 and 12). On top of controlling for sex in all analyses, we tested for interaction effects between sex and the number of negative and positive streamlines, since women were overrepresented in our sample (Supplement 13).

Due to the significantly negative correlation between CTQ and FSozU, we decided against including the variables as covariates in the respective predictive models. This would have artificially increased the model performance, as, within the CPM framework, a dependent variable is predicted by the connectome. If a variable (e.g., FSozU) highly correlated with this dependent variable (e.g., CTQ) were included as an additional predictor, the correlation between these two variables alone would result in better predictive performance of the model. However, this would not inform about the predictive utility of the connectome. Predicting FSozU and CTQ by the connectome only, we thus estimated the predictive utility of the connectome more accurately. However, we conducted a robustness check which verified that FSozU-connectome and CTQ-connectome associations remained significant when controlling for the respective other variable (Supplement 14). In addition, we performed analyses using one site as the training set and the other as the test set (Supplement 15), resulting in comparable model performance if the larger site serves as training set. Predictably, for the smaller site, the 45% decrease in training set size led to lower performance. We also analyzed the influence of current perceived stress as a potential proxy for current maltreatment, showing no impact of perceived stress or negative recent life events on NOS (Supplement 16).

## Discussion

To the best of our knowledge, this is the first study to identify structural brain networks which robustly and significantly predict retrospectively reported childhood maltreatment and currently perceived social support in a large sample of healthy adults. Importantly, the overlapping edges of maltreatment and social support represent a neurobiological signature of the interconnectedness of both constructs. Furthermore, while acknowledging the cross-sectional nature of these data, they tentatively suggest social support acts as a protective factor following adverse childhood experiences, compensating for brain network alterations. We implemented a repeated cross-validation scheme. This allowed us to thoroughly assess the stability of our results and quantify the predictive error of the predictive model but also for each edge. This can be considered an extension of the original CPM approach, as previous studies have used an arbitrary cut-off to identify stable edges. This extension of the original CPM approach might be necessary and useful for future studies, considering non-normally distributed predicted variables which are common for clinical and naturalistic studies.

For both constructs, positively and negatively associated networks were revealed. In general, hyperconnectivity should be considered as dysfunctional as hypoconnectivity, as it is not just a compensatory mechanism, but comes with a cost for network efficiency, and in the long run, can accelerate neurodegeneration through elevated metabolism [55].

The identification of the positive CTQ network between frontal, temporal and parietal regions implies that childhood maltreatment is not exclusively linked to decreased connectivity, as previous research suggests, but provides additional evidence for childhood maltreatment-related structural hyperconnectivity. Central hubs (i.e., nodes with the highest degree) were the pars triangularis, the inferior parietal, the pars opercularis, the precuneus, and the insula. Hyperconnectivity in the limbic system has been found previously in the context of functional connectivity [3, 56] and possibly reflects a heightened alertness and threat response in individuals who, based on early experiences, perceive social interactions, amongst others, as more critical [57]. Overlapping findings of structural and functional hyperconnectivity are in line with findings showing a general overlap of structural and functional connectivity [58, 59], supporting the view that structural networks provide an anatomical constraint for functional networks [60, 61]. Nonetheless, given that there is not a one-to-one but rather a one-to-many relationship between structural and functional connections, these two constructs should not be equated [62–64].

The network negatively associated with CTQ was mostly constrained to frontal regions, with the left rostral middle frontal gyrus (RMF), a part of the dorsolateral prefrontal cortex (dlPFC) as the most dominant hub. With increasing maltreatment scores, significantly lower connectivity was found between the RMF and other hubs, such as the insula and the superior frontal and middle temporal gyrus. Decreased connectivity in frontal areas, which have previously been associated with childhood maltreatment [12, 65] but also self-referential processing [66, 67], might relate to a difficulty of understanding others’ intentions in social situations. The inverse correlation between FSozU and CTQ was mainly driven by the CTQ subscale emotional neglect. This suggests that early learning experiences might have resulted in the negligence of emotional attachments and/or the development and maintenance of dysfunctional relationship schemes.

Networks which were positively associated with social support were mainly located in frontal and parietal regions, including the precentral and postcentral lobe, the precuneus and the superior frontal lobe. This is in line with theories about the ‘social brain’ attributed to the frontal neocortex of the brain [68, 69]. In the negative social support network, hubs in the frontal, parietal and temporal regions played a role. This is partly in contrast to previous literature: increased structural integrity of white matter tracts in the frontal and temporal cortex has been revealed with increasing social network size [31] and social network diversity [30]. However, while network size is a rather quantitative measure of social support, the measure we used here is a broader measure of the subjective perception of the quality of the relationships, encompassing friendship, family, but also colleagues and neighbors.

This is the first CPM study predicting not only the phenotype of two measures separately, but also statistically evaluating shared edges of two constructs. Concerning the network which was positively associated with childhood maltreatment, but negatively with social support, shared edges with a high stability (> 90%) connected temporal areas (e.g., the lingual gyrus and temporal pole), but also frontal and parietal areas (e.g., precentral and superior temporal gyrus). Alterations in temporal regions, e.g., in hippocampal gray matter volume have been reported in maltreated vs. non-maltreated individuals, whereas social support acted as a moderating factor [70]. In the network negatively associated with childhood maltreatment but positively with social support, most stable edges were concentrated in frontal, anterior temporal, and parietal areas.

The fact that all edges which were positively associated with childhood maltreatment were in turn negatively associated with increasing social support, and vice versa, has several implications.

First, in the present study the interrelation of networks associated with social support and maltreatment experiences resembles their negative correlation at the behavioral level. Indeed, research has documented very well how childhood maltreatment influences social functioning [71, 72] and the perception of social interactions later in life mediated by altered threat, reward and emotion processing [73]. On the one hand, experiences of childhood maltreatment influence statements about perceived social support [74, 75]. Maltreated children are hypervigilant to potential threats which can lead to withdrawal [57, 76]. While being functional in the short term, this increased alertness represents a maladaptive strategy in the long run that might increase the risk for psychopathology. Moreover, childhood maltreatment leads to more peer victimization as children learned early that social interactions are rather negative and potentially harmful [74]. On the other hand, resilience might also flourish on the individual ability to recruit social support as a strategy to maintain good mental health.

Second, following this, these opposed effects might be indicative of a moderating, more specifically, protective effect of social support. Network alterations associated with childhood maltreatment could be attenuated with increasing perceived social support. In the same way, the neurocognitive social transactional model presents two alternative outcomes of childhood maltreatment [75]: if there is sufficient protective influence such as social support, the neurocognitive adaptations can lead to adjustment or compensation in neurocognitive functioning. However, if protective factors are scarce, social thinning can be a consequence and renders the individual even more vulnerable to negative social interactions. Indeed, maltreatment-related disruption might be reversible, as suggested by normalizing white matter integrity in children from harmful family backgrounds after placing them in nurturing foster homes [77]. This corroborates our assumption that social support can interact with the diminishing effects of childhood maltreatment on connectivity. On a molecular level, this might be explained by effects of oxytocin, a neuropeptide produced in the hypothalamus, which has been linked to cellular anti-aging effects [78, 79], and reduction of stress due to social isolation [80], especially in the presence of negative childhood experiences [81]. Psychological stress and constant threats induced by childhood maltreatment can be viewed as chronic inflammatory stimuli that affect the corresponding brain regions and circuits, e.g., the amygdala [82], and can damage white matter [83]. Importantly, research has also focused on anti-inflammatory and anti-oxidant impacts of oxytocin on the brain [84] and its contribution to neurite growth and formation of neural circuits [85]. Oxytocin most likely interacts with microglial cells and astrocytes which are both important agents in brain neuroinflammatory processes [83]. Moreover, low coping self-efficacy, including social support, was previously associated with elevated inflammatory cytokines [86]. Therefore, social support might exert its neuroprotective role on white matter-damaging effects of maltreatment by the associated release of oxytocin. Facing the negative correlation of social support and CTQ, as well as the large contribution of social support on mental health, clinicians should focus on empowering their patients to build and strengthen social networks and support systems, e.g., by commonly implemented social skills training during psychotherapy. Furthermore, a novel ‘psychobiological therapy’ approach, i.e., a combination of psychotherapy with administration of oxytocin (receptor agonizts) may be suitable [87]. Longitudinal studies could examine the dynamic changes in brain networks over time and explore how interventions targeting social support may modulate these networks. Additionally, investigating the impact of therapeutic interventions on brain connectivity could provide further evidence for the clinical relevance of our findings.

The present sample comprised participants without clinically relevant depressive symptoms. While the inclusion of a sample with depressed participants would have increased the variance of CTQ and FSozU scores, we intentionally chose a healthy sample to avoid confounding effects of mental illnesses including social withdrawal, symptom severity, medication and a potential bias in self-report measures (Supplement 17). Nevertheless, future studies should investigate our results in participants with mental disorders. Possibly, our participants with relatively high CTQ scores might therefore be considered resilient, as they have maintained good mental health despite experiencing early potentially traumatic events which is also reflected in high self-reported social functioning. The effect sizes observed in this study were rather small, however, they were within the range expected by Shen et al. [33]. Moreover, small effect sizes are common in large samples since they have proven to deliver the true effect size more precisely as compared to small samples which are most likely to provide inflated effect sizes [88].

### Limitations

Due to the cross-sectional nature of our data we are not able to draw conclusions about causality. Nonetheless, since childhood maltreatment experiences were enquired and naturally happened before the perception of current social support, we assume that in this sample, maltreatment most likely predicted social support and rather not vice versa.

While we reviewed evidence that the perception of social interactions is confounded by childhood maltreatment, the positive, evaluative wording of the items of the social support questionnaire (e.g., “I have a familiar person who I feel very comfortable around.”) supports the interpretation of the collected construct as an actual protective factor as opposed to the perception of stressful social proximity.

Although retrospectively self-reported experience of child maltreatment is often discussed in previous literature due to a possible depressive bias and/or false memories [89], a longitudinal study showed that the CTQ has a high temporal stability across various mood states [43]. Unfortunately, we did not have any measure neither of current maltreatment nor of timing of CM. Further studies are needed to consolidate the findings using structured clinical expert interviews, e.g., the MACE [90]. Since we only screened for Axis-I disorders of the DSM-IV-TR, we cannot rule out the possibility that a few individuals met the criteria for a personality disorder. Our analyses were based on connectivity matrices that were weighted by the number of reconstructed white matter streamlines. While this measure provided superior prediction accuracies of phenotypal data in previous studies [91], it, nevertheless, represents an indirect measure of structural connectivity [92].

## Conclusion

In a large sample of healthy adults, we identified networks which robustly predict childhood maltreatment and social support from structural connectome data. Importantly, we highlight inverse effects of social support on maltreatment-related structural connectivity alterations, most prominently in frontal and temporal regions. The current work reiterates the importance of social support, not only on the previously studied behavioral level, but also on a neural network-level, thus contributing to the understanding of neurobiological mechanisms underlying the protective effect of good social health. Our work illustrates and underlines the importance of fostering and sustaining beneficial social relationships in individuals who experienced maltreatment during childhood.

### Supplementary information

Supplemental Material

## Data Availability

Analyses were performed in Matlab 2019b. Codes will be available upon request and communication with the corresponding author.
