# Supplementary material for: Shared and distinct structural brain networks related to childhood maltreatment and social support: connectome-based predictive modeling
Source: Mol Psychiatry. 2023 Sep 15;28(11):4613–21. doi: 10.1038/s41380-023-02252-3 (PMC10914611; doi:10.1038/s41380-023-02252-3)
Supplement: Supplementary file 1 — Supplemental Material [file 41380_2023_2252_MOESM1_ESM.docx]

# Supplementary Material 1: Choosing participants from the FOR2107 cohort

Data without dropouts
*N* = 945 HC

Total Sample

*N* = 904 HC

3^rd^ datafreeze (May 2022)

*N* = 2479

Dropout of missing data
(*N* = 21) or participants with lifetime Axis I disorders
(*N* = 1409)

Total data
*N* = 1049 HC

Clinical dropout criteria (e.g., severe somatic illness, MRI contraindication) (*N* = 104)

MRI Data not surviving quality checks

T1: *N* = 13

DTI: *N* = 26

Connectome: *N* = 1

The third data freeze (May 11^th^, 2022) from the FOR2107 cohort was used comprising *N* = 2478 participants, of which participants with Axis I diagnoses (e.g., Major Depressive Disorder, Bipolar Disorder, Schizophrenia, substance dependence/abuse), or missing data, were excluded leaving a sample of *N* = 1049. In a next step, dropout criteria such as severe somatic illness, traumatic brain injury or MRI contraindications were applied which led to a basic sample of *N* = 945. After implementing quality checks of the MRI data (DTI & T1), the final sample consisted of *N* = 904 healthy controls (HC). Severe neurological illnesses, such as brain tumors, traumatic brain injuries, or a history of stroke, were excluded, leaving only participants in the sample whose somatic diseases do not influence affect, require current, acute treatment, produce brain alterations or complicate participation (e.g., tremor). Furthermore, we only included participants with Caucasian ancestry recruited from two West German university cities. Therefore, our results cannot be generalized to other populations and geographic regions.

The DTI data from the above participants have already been used partly in previously published, topic-related studies [1–3].

The FSozU, including its short form (F-SozU K-6; [4], is widely accepted for assessing general social support in German-speaking countries and in cross-cultural contexts [5, 6], both in the general population and in clinical trials [4] Numerous previous studies have investigated the FSozU, examining its role as a protective factor, such as in a large-scale longitudinal study investigating resilience factors [7, 8] and as a mediator between CM and depressive symptoms [9]. Neurologically, social support as measured by the FSozU was previously positively associated with white matter fibers [10] and negatively with gray matter in individuals who have experienced CM [11].

# Supplementary Material 2: MRI data acquisition & preprocessing

## MRI acquisition details

Both T1 and DTI data were acquired using a 3T whole body MRI scanner (Marburg: Tim Trio, 12-channel head matrix Rx-coil, Siemens, Erlangen, Germany; Münster: Prisma, 20-channel head matrix Rx-coil, Siemens, Erlangen, Germany). A GRAPPA acceleration factor of two was employed for both sequences. A high resolution T1-weighted dataset were acquired using a 3D-MPRAGE-sequence (TE_Münster_ = 2.28 ms, TE_Marburg_ = 2.26 ms, TR_Münster_ = 1900 ms, TR_Marburg_ = 2130 ms TI = 900 ms) with an isotropic voxel size of 1 x 1 x 1 mm³.  For DTI imaging, fifty-six axial slices with no gap were measured with an isotropic voxel size of 2.5 x 2.5 x 2.5 mm³ (TE = 90 ms, TR = 7300 ms). Five non-DW images (b = 0 s/mm²) and 2 x 30 DW images with a b-value of 1000 s/mm² were acquired. The second set was used to average across both acquisitions to increase signal-to-noise-ratio and detect artifacts. Imaging pulse sequence parameters were standardized across both sites to the extent permitted by each platform. Besides the bi-centric collection of MRI data, a body coil had to be exchanged in Marburg in the course of our study. Therefore two dummy-coded variables (Marburg pre body-coil: yes/no, Marburg post body-coil: yes/no) with Münster as reference category were created to correct for site and equipment exchange [12]. Visual inspection of structural images (T1 and Freesurfer for surface) led to exclusion in case of anatomical abnormalities. Moreover, a phantom was measured regularly at the MRI to ensure consistent and accurate calibration of the imaging equipment. It was statistically evaluated by a dedicated work package of the MACS (WP6) [12]. In addition, participants’ heads were carefully padded in the head coil to minimize head motion.

## Preprocessing of diffusion-weighted images

Diffusion-weighted images (DWI) were realigned and corrected for eddy currents and susceptibility distortions [13] using FSL's eddy (Version 6.0.1). Diffusion tensor imaging models the measured signal of a voxel by a single tensor describing the diffusion signal as one preferred diffusion direction per voxel. The CATO toolbox employed for reconstruction of the anatomical connectome uses the informed RESTORE algorithm [14, 15] that estimates the tensor while identifying and removing outliers during the fitting, thereby reducing the impact of physiological noise artifacts on the DTI modeling.

## Anatomical connectome reconstruction

We employed the publicly available [CATO toolbox](http://www.dutchconnectomelab.nl/CATO/) for reconstructing the anatomical connectome. The procedure included the following steps:

We obtained a network of 114 brain regions along with the reconstructed white matter streamlines between these brain areas for each participant. To identify the brain areas, we relied on FreeSurfer's Desikan-Killiany Atlas [16–18]. However, given the poorer DWI signal-to-noise ratio in subcortical regions and the dominant effect of subcortical regions on network properties, we decided to use a subdivision of this atlas containing only cortical regions, as we have done in previous work [19, 20].

To reconstruct the streamlines, we applied a deterministic streamline tractography based on the Fiber Assignment by Continuous Tracking (FACT) algorithm [21]. We chose this deterministic algorithm instead of more advanced diffusion direction reconstruction methods because it provides a reasonable balance between false-negative and false-positive fiber reconstructions [22]. Connections between two nodes, i.e., brain areas, were included if at least three reconstructed streamlines connected them. Edges were assigned to all nodes it traverses or ends in. This type of thresholding was applied since we wanted to balance the sensitivity and specificity of the resulting connectivity matrices [23, 24]. Additionally, we applied one-tailed one sample *t*-tests to each edge and included only those edges that deviated significantly (*p* < .05) from zero across all participants. This step was included to retain only highly consistent edges, as has been done in previous connectome studies (e.g., [25]).

Each participant's network was finally stored in a connectivity matrix with rows and columns representing nodes and matrix entries representing edges (i.e., connectivity strength, measured as the number of reconstructed streamlines (NOS) between two nodes).

# Supplementary Material 3: Quality control of connectivity matrices

Measures for outlier detection included 1. average number of streamlines, 2. average fractional anisotropy, 3. average prevalence of each subject's connections (low value, if the subject has "odd" connections), and 4. average prevalence of each subjects connected brain regions (high value, if the subject misses commonly found connections). For each metric the quartiles (Q1, Q2, Q3) and the interquartile range (IQR = Q3-Q1) was computed across the group and a datapoint was declared as an outlier if its value was below Q1-1.5*IQR or above Q3+1.5*IQR on any of the four metrics.

# Supplementary Material 4: Testing reciprocal relationships

**Predicting CTQ networks from FSozU**

**Figure 1**


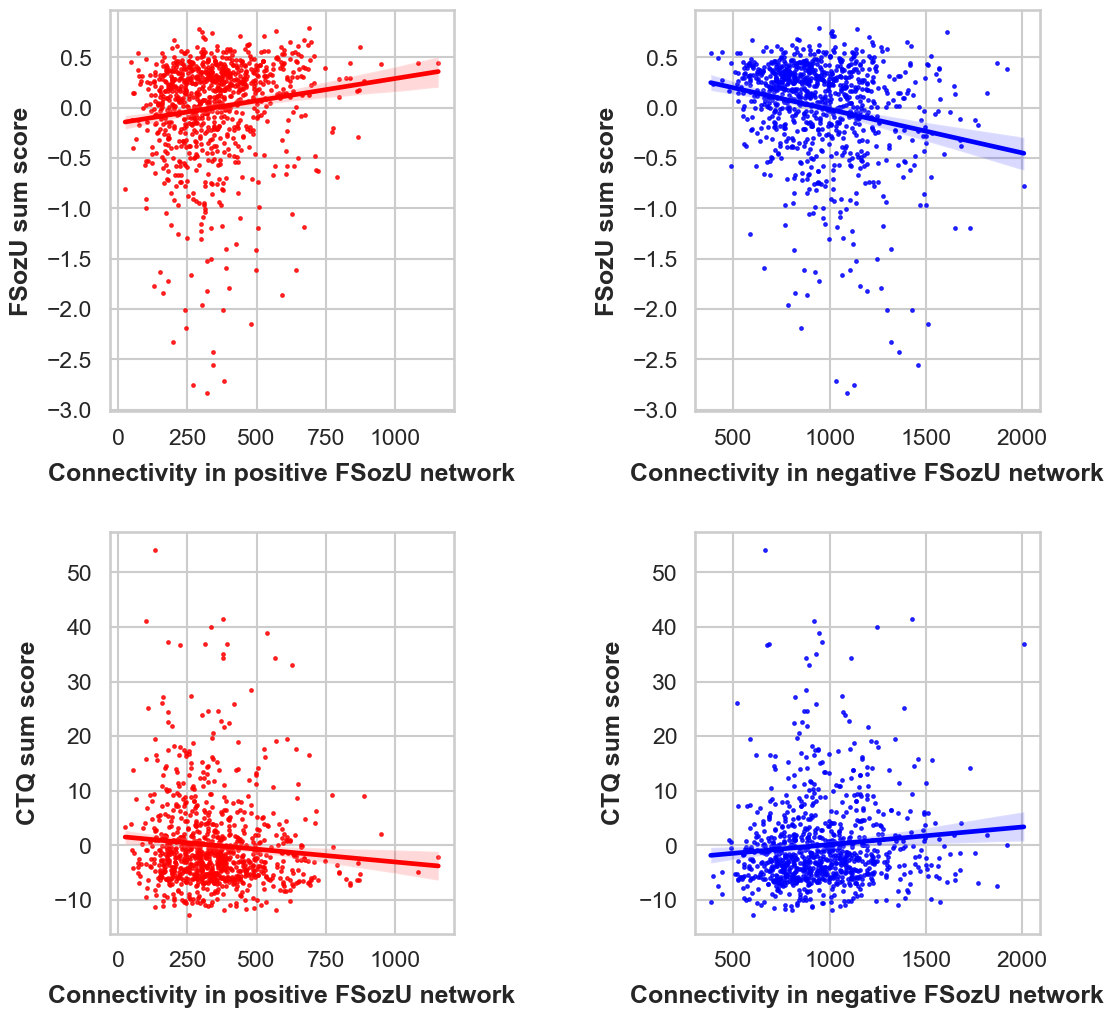
 *Associations of CTQ and the connectivity strength in positive and negative FSozU networks*

**Table 1**

*Comparing coefficients of the model predicting FsozU from CTQ and models additionally correcting for CTQ*

| Model | | Estimate | | *SE* | | ***t*** | | *P* > \|*t*\| | | 97.5% CI | | | |
| --- | --- | --- | --- | --- | --- | --- | --- | --- | --- | --- | --- | --- | --- |
|  | |  | |  | |  | |  | | *LL* | | *UL* | |
| CTQ ⁓ FSozU | |  | |  | |  | |  | |  | |  | |
| Positive (sumpos) | | -0.0940 | | 0.034 | | -2.791 | | .005 | | -.160 | | -.028 | |
| Negative (sumneg) | | 0.1090 | | 0.035 | | 3.138 | | .002 | | .041 | | .177 | |
| Corrected for FSozU (sumpos) | | -0.0443 | | .032 | | -1.386 | | .166 | | -.107 | | .018 | |
| Corrected for FSozU (sumneg) | | 0.0270 | | 0.034 | | 0.805 | | .421 | | -.039 | | .093 | |

**Predicting FSozU networks from CTQ**

**Figure 2**

*Associations of FSozU and the connectivity strength in positive and negative CTQ networks*


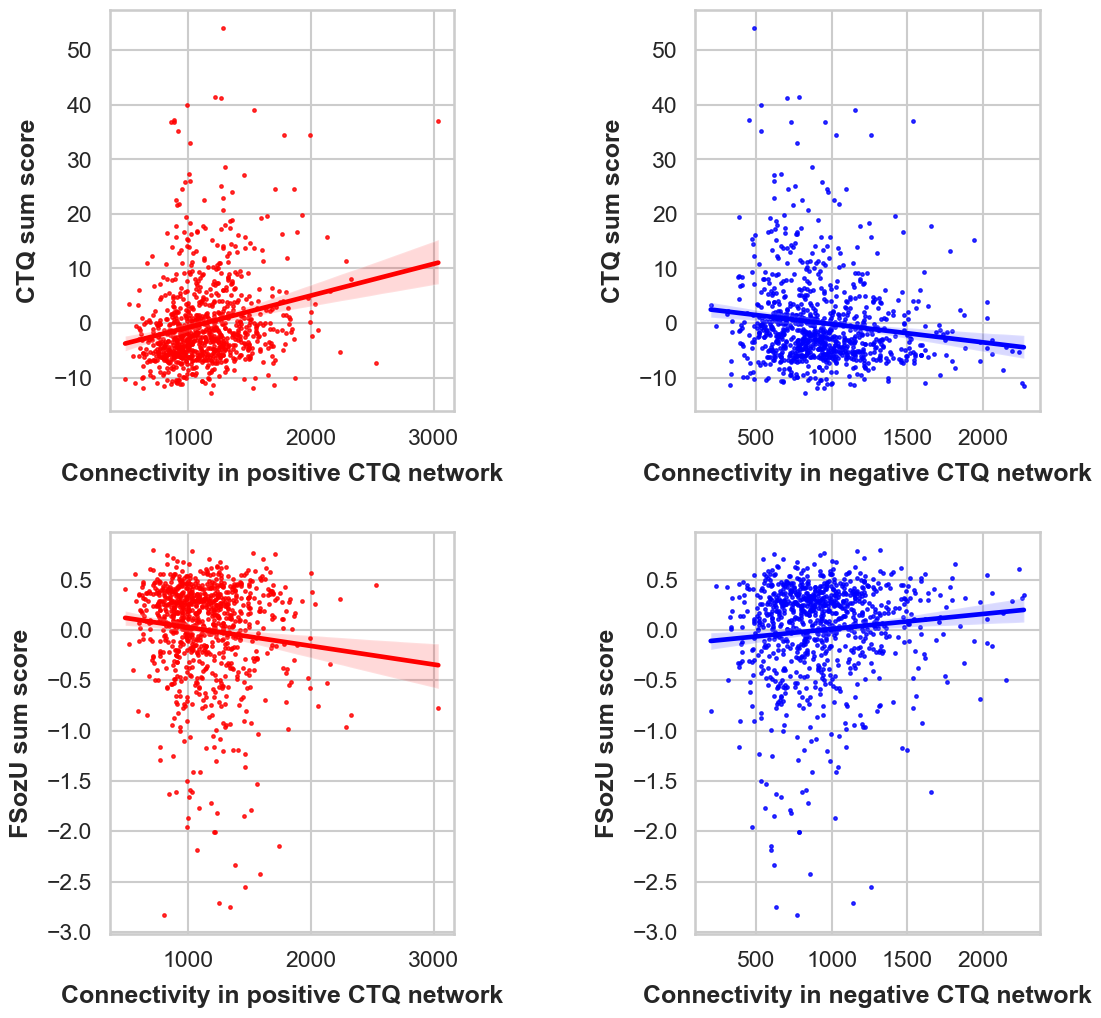


**Table 2**

*Comparing coefficients of the model predicting FsozU from CTQ and models additionally correcting for CTQ*

| Model | | Estimate | | *SE* | | ***t*** | | *P* > \|*t*\| | | 97.5% CI | | | |
| --- | --- | --- | --- | --- | --- | --- | --- | --- | --- | --- | --- | --- | --- |
|  | |  | |  | |  | |  | | *LL* | | *UL* | |
| FSozU ⁓ CTQ | |  | |  | |  | |  | |  | |  | |
| Positive (sumpos) | | -0.1211 | | 0.035 | | -3.489 | | .001 | | -0.189 | | -0.053 | |
| Negative (sumneg) | | 0.1013 | | 0.034 | | 2.988 | | .003 | | .035 | | .168 | |
| Corrected for CTQ (sumpos) | | -0.0392 | | 0.034 | | -1.170 | | .242 | | -.105 | | .027 | |
| Corrected for CTQ (sumneg) | | 0.0525 | | 0.032 | | 1.631 | | .103 | | -.011 | | .116 | |

# Supplementary Material 5: Correlation of FSozU and CTQ subscales

**Table 3**

*Bivariate Pearson’s* r *between FSozU and CTQ subscale scores*

| Variable | *N* | 1 |
| --- | --- | --- |
| 1. Perceived social support (FSozU) | 904 | - |
| 2. CTQ_Sum score | 904 | -.370** |
| 3. CTQ_Physical abuse | 904 | -.163** |
| 4. CTQ_Emotional abuse | 904 | -.264** |
| 5. CTQ_Sexual abuse | 904 | -.073** |
| 6. CTQ_Physical neglect | 904 | -.248** |
| 7. CTQ_Emotional neglect | 904 | -.444** |

*Note.* ***p*<.001

**Table 4**

*Linear regression testing the association between CTQ subscales and positive and negative CTQ networks*

| Model | Estimate | 95% CI | | *SE* | Estimate (β) | *T* | *p* |
| --- | --- | --- | --- | --- | --- | --- | --- |
|  |  | *LL* | *UL* |  |  |  |  |
| Positive |  |  |  |  |  |  |  |
| CTQ_Physical abuse | 2.958 | -12.233 | 18.148 | 7.740 | 0.015 | 0.382 | .702 |
| CTQ_Emotional abuse | 3.819 | -5.426 | 13.064 | 4.710 | 0.036 | 0.811 | .418 |
| CTQ_Sexual abuse | 8.092 | -5.596 | 21.780 | 6.974 | 0.038 | 1.160 | .246 |
| CTQ_Physical neglect | 7.018 | -4.937 | 18.973 | 6.091 | 0.043 | 1.152 | .250 |
| CTQ_Emotional neglect | 12.346 | 5.183 | 19.509 | 3.650 | 0.150 | 3.383 | <.001 |
| Age | -1.226 | -2.804 | 0.352 | .804 | -0.052 | -1.525 | .128 |
| Head motion | -750.291 | -1689.175 | 188.594 | 478.382 | -0.092 | -1.568 | .117 |
| Sex | -28.784 | -78.556 | 20.988 | 25.360 | -0.045 | -1.135 | .257 |
| Bcpre | 201.304 | 125.395 | 277.213 | 38.677 | 0.322 | 5.205 | <.001 |
| Bcpost | 175.629 | 106.054 | 245.205 | 35.450 | 0.229 | 4.954 | <.001 |
| TIV | 0.605 | 0.438 | 0.772 | 0.085 | 0.287 | 7.114 | <.001 |
| Negative |  |  |  |  |  |  |  |
| CTQ_Physical abuse | -1.353 | -18.644 | 15.939 | 8.810 | -0.006 | -0.154 | .878 |
| CTQ_Emotional abuse | -10.917 | -21.440 | -0.394 | 5.362 | -0.094 | -2.036 | .042 |
| CTQ_Sexual abuse | -7.461 | -23.042 | 8.120 | 7.939 | -0.032 | -0.940 | .348 |
| CTQ_Physical neglect | -2.797 | -16.405 | 10.811 | 6.934 | -0.016 | -0.403 | .687 |
| CTQ_Emotional neglect | -2.519 | -10.673 | 5.634 | 4.154 | -0.028 | -0.606 | .544 |
| Age | -0.114 | -1.910 | 1.682 | 0.915 | -0.004 | -0.124 | .901 |
| Head motion | -629.801 | -1698.525 | 438.922 | 544.537 | -0.070 | -1.157 | .248 |
| Sex | 32.455 | -24.200 | 89.110 | 28.867 | 0.047 | 1.142 | .261 |
| Bcpre | -15.958 | -102.364 | 70.448 | 44.026 | -0.023 | -0.362 | .717 |
| Bcpost | 34.711 | -44.486 | 113.907 | 40.352 | 0.042 | 0.860 | .390 |
| TIV | 0.664 | 0.474 | 0.854 | 0.097 | 0.288 | 6.863 | <.001 |

*Note.* Abbreviations: CI=confidence interval; LL=lower limit; UL=upper limit; SE=standard error; CTQ=childhood trauma questionnaire, Bcpre=dummy-coded variable correcting for a body coil exchange in Marburg; Bcpost= dummy-coded variable correcting for a body coil exchange in Marburg; TIV=total intracranial volume;

# Supplementary Material 6: Confirmatory factor analysis of CTQ and FSozU

We conducted a confirmatory factor analysis with a two-factor solution for the CTQ and FSozU. To this end, we used 25 CTQ items, i.e., without the three Minimization Scale items, and all 22 FSozU items. The Kaiser–Meyer–Olkin measure of sampling adequacy was .934, representing a relatively good factor analysis, and Bartlett’s test of Sphericity was significant (*p*<.001), indicating that correlations between items were sufficiently large for performing a factor analysis. As extraction method, we used Maximum Likelihood and Direct Oblimin since we expected the factors to be correlated. This revealed that both questionnaires were mainly correlated with their individual construct (namely, childhood maltreatment (CM) and social support). The only items which were correlated with social support and CM, were the five items from the subscale *emotional neglect*. This matches the observation that this subscale shows the highest bivariate correlation with the FSozU (Supplement 5).

**Table 5**

*Results from a confirmatory factor analysis of CTQ and FSozU items*

| Item | Factor loading | |  |
| --- | --- | --- | --- |
|  | 1 | 2 | |
| Factor 1: Childhood Maltreatment |  |  | |
| CTQ 1 “I did not have enough to eat.” | .076 | -.025 | |
| CTQ 2 “Someone protected me.” (R) | .253 | -.189 | |
| CTQ 3 “Someone in my family called me stupid, lazy or ugly.” | .297 | -.170 | |
| CTQ 4 “My parents were too high to care for my family.” | .182 | -.118 | |
| CTQ 5 “Someone made me feel special.” (R) | .228 | **-.363** | |
| CTQ 6 “I had to wear dirty clothes.” | .196 | -.077 | |
| CTQ 7 “I had the feeling of being loved.” (R) | .282 | **-.384** | |
| CTQ 8 “I thought my parents wished I was never born.” | **.318** | -.158 | |
| CTQ 9 “I was beat so badly I had to go to hospital.” | .255 | -.060 | |
| CTQ 11 “Someone beat me so badly I had bruises.” | **.322** | -.073 | |
| CTQ 12 “I was punished with a belt, stick, or hard object.” | .291 | -.129 | |
| CTQ 13 “My family members cared for each other.” (R) | .293 | **-.325** | |
| CTQ 14 “My family called me names or insulted me.” | .292 | -.214 | |
| CTQ 15 “I think I was physically abused when I grew up.” | **.530** | .059 | |
| CTQ 17 “I was beat so badly that someone noticed (teacher, doctor).” | .226 | -.087 | |
| CTQ 18 “I had the feeling someone in my family hated me.” | .289 | -.136 | |
| CTQ 19 “My family members felt close to each other.” (R) | .269 | **-.336** | |
| CTQ 20 “Someone tried to touch me sexually or made me touch them.” | **.940** | .250 | |
| CTQ 21 “Someone threatened me to hurt me or tell lies about me, if I did not have sexual interactions with them.” | **.706** | .213 | |
| CTQ 23 “Someone tried to make me do sexual things or watch them.” | **.905** | .262 | |
| CTQ 24 “Someone molested me sexually.” | **.959** | .279 | |
| CTQ 25 “I think I was emotionally abused when I grew up” | **.543** | -.006 | |
| CTQ 26 “Someone brought me to the doctor when needed.” (R) | .248 | -.139 | |
| CTQ 27 “I think I was sexually abused when I grew up.” | **.900** | .276 | |
| CTQ 28 “My family was a source of support.” (R) | **.341** | **-.355** | |
| Factor 2: Social support |  |  | |
| FSozU 1 “I have people who can look after my apartment (flowers, pets) when I’m not there” | .051 | **.434** | |
| FSozU 2 “There are people who take me as I am.” | -.016 | **.637** | |
| FSozU 3 “My friends or relatives care to hear my opinion.” | .044 | **.625** | |
| FSozU 4 “I want more affection and support from others.” (R) | -.042 | .257 | |
| FSozU 5 “I have a very familiar person whose help I can count on.” | .039 | **.687** | |
| FSozU 6 “I can rent groceries and tools when needed.” | .008 | **.500** | |
| FSozU 7 “I have friends and relatives who can listen to me when I need to talk.” | .055 | **.745** | |
| FSozU 8 “I almost don't know anyone I like to go out with.” (R) | .009 | **.424** | |
| FSozU 9 “I have friends or relatives who I can just give a hug.” | .041 | **.693** | |
| FSozU 10 “When I’m sick, I can ask friends/relatives without hesitating to do important errands for me.” | -.002 | **.656** | |
| FSozU 11 “When I’m deeply sad, I know who to turn to.” | .056 | **.779** | |
| FSozU 12 “I often feel like an outsider.” (R) | -.106 | **.367** | |
| FSozU 13 “There are people who share joy and misery with me.” | .066 | **.813** | |
| FSozU 14 “With some friends/relatives I can be quite boisterous at times.” | .052 | **.765** | |
| FSozU 15 “I have a trusted person who I feel very comfortable around.” | .006 | **.704** | |
| FSozU 16 “I have enough people who really help me out when I’m stuck.” | -.024 | **.809** | |
| FSozU 17 “There are people who are on my side even when I make mistakes.” | .024 | **.787** | |
| FSozU 18 “I want more security and proximity.” (R) | -.068 | .228 | |
| FSozU 19 “There are enough people who I have a really good relationship with.” | .025 | **.715** | |
| FSozU 20 “There is a community of people (friends, clique) who I feel close to.” | .001 | **.632** | |
| FSozU 21 “From my friends and acquaintances, I often receive good advice (good doctor, important information).” | .035 | **.622** | |
| FSozU 22 “There are people who I can show all my emotions without feeling embarrassed.” | .046 | **.758** | |

*Note.* *N* = 904. The extraction method was Maximum Likelihood with an oblique (Oblimin direct with Kaiser Normalization) rotation. Factor loadings above .30 are in bold. Reverse-scored items are denoted with an (R).

# Supplementary Material 7: Detailed description of networks: CTQ

**Table 6**

*Negative network hubs CTQ*

| Node | Degree |
| --- | --- |
| ctx-lh-rostralmiddlefrontal_3 | 3 |
| ctx-rh-superiorparietal_2 | 3 |
| ctx-lh-lateraloccipital_1 | 3 |
| ctx-lh-pericalcarine_1 | 2 |
| ctx-lh-isthmuscingulate_1 | 2 |
| ctx-rh-superiorfrontal_3 | 2 |
| ctx-rh-posteriorcingulate_1 | 2 |
| ctx-rh-lateraloccipital_2 | 2 |

*Note.* The listed hubs are the top 15 % of nodes with the highest degree within the negative CTQ network. Abbreviations: lh = light hemisphere; rh = right hemisphere

**Table 7**

*Positive network hubs CTQ*

| Node | Degree |
| --- | --- |
| ctx-lh-parstriangularis_1 | 4 |
| ctx-lh-inferiorparietal_2 | 4 |
| ctx-rh-parsopercularis_1 | 4 |
| ctx-lh-precuneus_1 | 4 |
| ctx-rh-insula_1 | 4 |
| ctx-rh-superiorparietal_1 | 3 |
| ctx-rh-precuneus_2 | 3 |
| ctx-lh-medialorbitofrontal_1 | 3 |
| ctx-rh-entorhinal_1 | 2 |

*Note.* The listed hubs are the top 15 % of nodes with the highest degree within the positive CTQ network. Abbreviations: lh = light hemisphere; rh = right hemisphere

**Figure 3**

*Associations of CTQ and the connectivity strength in positive and negative networks – original model*

**
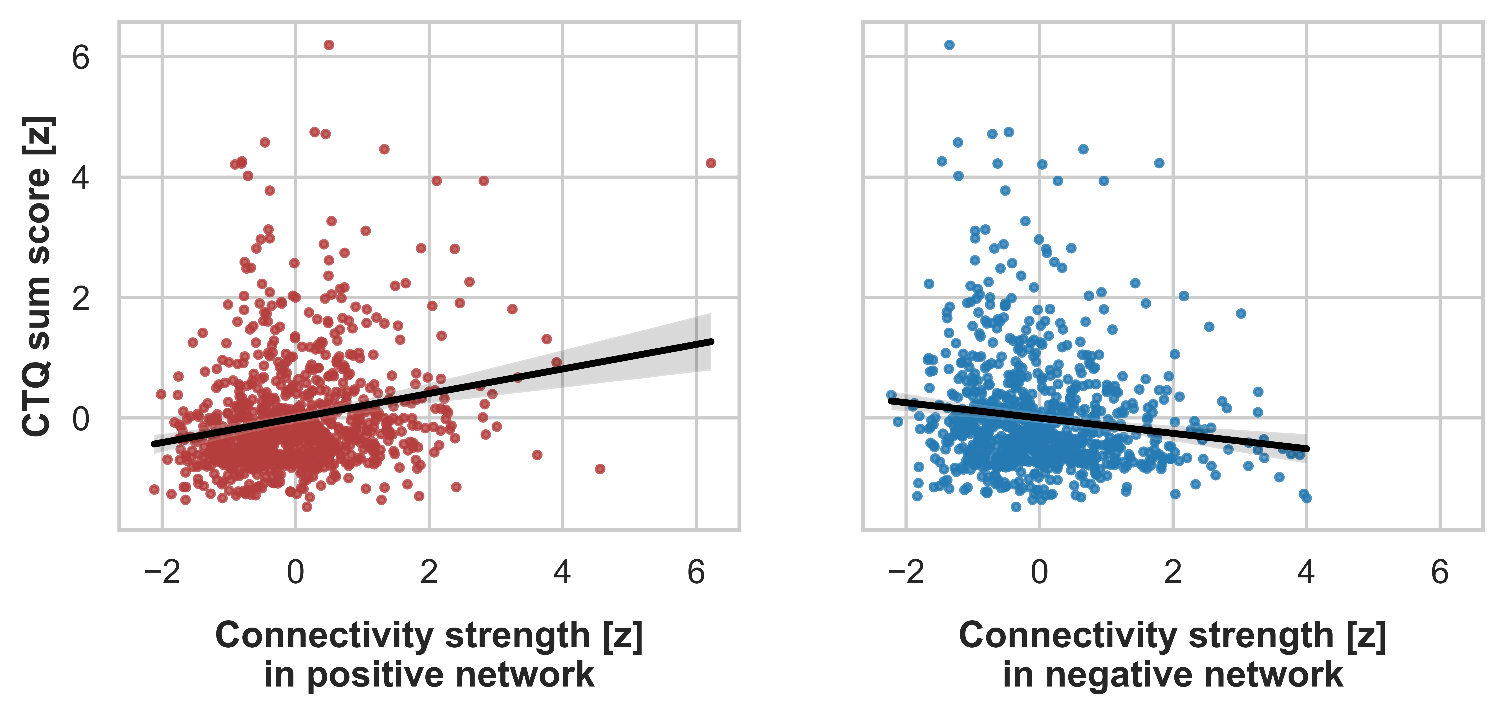
**

*Note.* The figure shows the association between the CTQ sum score and the connectivity strength (i.e., the total number of streamlines) of all edges that had a significant edge stability and were either positively (“positive network”) or negatively (“negative network”) associated to the CTQ. Note that CTQ values were corrected for influences of age, head motion, sex, total intracranial volume, site which can result in negative values.

**Figure 4**

*Figure 3 without multivariate outliers identified according to Mahalanobis distance*

**
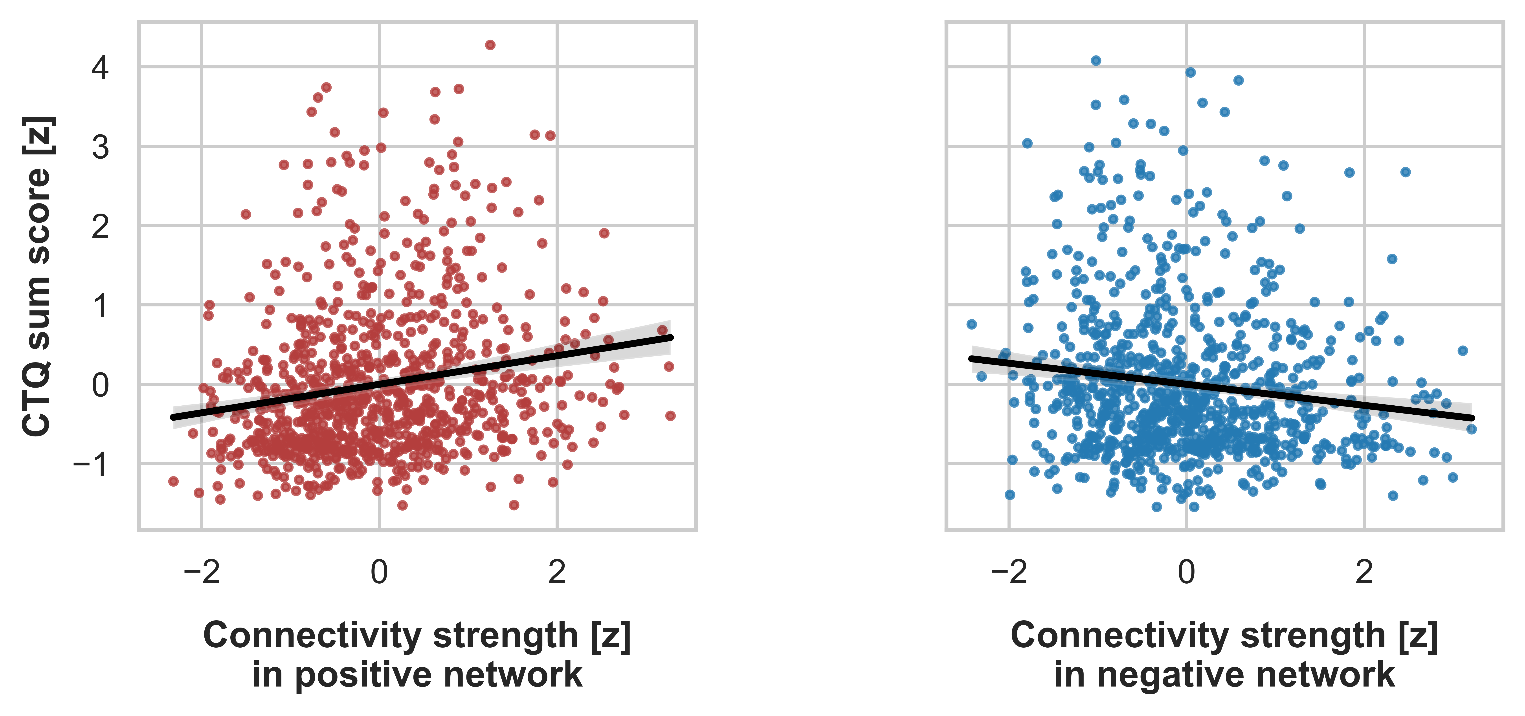
**

**Figure 5**

*Heatmaps of the regional distribution of brain regions*

*
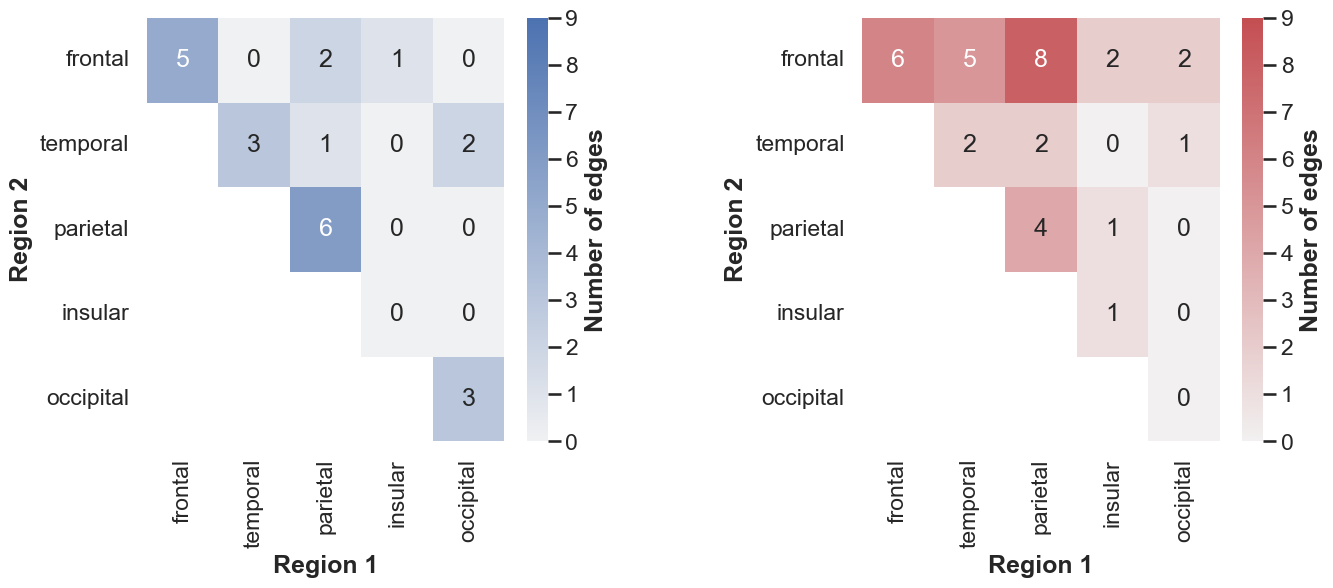
*

*Note.* The figure shows the number of network edges from the CTQ networks that connect frontal, temporal, parietal, and occipital brain regions. Red shows positive networks, blue negative networks.

**Table 8**

*Stability of edges associated with CTQ, and involved nodes*

| **Edge Correlation** | **Edge Stability (abs.)** | **Node 1** | **Node 2** | **Region 1** | **Regio n2** | ***p*_EdgeStability_** |
| --- | --- | --- | --- | --- | --- | --- |
| -0,109200639 | 1 | ctx-lh-postcentral_1 | ctx-lh-precentral_2 | parietal | frontal | 0 |
| -0,115900504 | 1 | ctx-lh-rostralmiddlefrontal_3 | ctx-lh-superiorparietal_3 | frontal | parietal | 0 |
| -0,111232388 | .999 | ctx-lh-lateralorbitofrontal_2 | ctx-lh-parsorbitalis_1 | frontal | frontal | 0 |
| -0,101032616 | .997 | ctx-lh-entorhinal_1 | ctx-lh-temporalpole_1 | temporal | temporal | .001 |
| 0,095434882 | .995 | ctx-lh-parstriangularis_1 | ctx-lh-pericalcarine_1 | frontal | occipital | .003 |
| -0,124028101 | .994 | ctx-rh-postcentral_2 | ctx-rh-posteriorcingulate_1 | parietal | parietal | .003 |
| 0,095373112 | .982 | ctx-lh-inferiorparietal_1 | ctx-lh-inferiorparietal_2 | parietal | parietal | .006 |
| -0,095387379 | .977 | ctx-lh-rostralmiddlefrontal_3 | ctx-lh-insula_1 | frontal | insular | .003 |
| 0,090291848 | .968 | ctx-rh-cuneus_1 | ctx-rh-precuneus_1 | occipital | parietal | .004 |
| -0,090733461 | .966 | ctx-lh-lateraloccipital_1 | ctx-lh-rostralmiddlefrontal_3 | occipital | frontal | .007 |
| 0,088494317 | .958 | ctx-lh-inferiorparietal_2 | ctx-lh-middletemporal_1 | parietal | temporal | .006 |
| 0,088132139 | .952 | ctx-rh-parsopercularis_1 | ctx-rh-superiortemporal_2 | frontal | temporal | .008 |
| -0,088107775 | .943 | ctx-lh-inferiortemporal_1 | ctx-lh-lateraloccipital_1 | temporal | occipital | .004 |
| -0,08906478 | .942 | ctx-rh-caudalmiddlefrontal_1 | ctx-rh-precentral_2 | frontal | frontal | .009 |
| -0,085201358 | .918 | ctx-rh-fusiform_2 | ctx-rh-temporalpole_1 | temporal | temporal | .01 |
| 0,084024508 | .908 | ctx-lh-precentral_1 | ctx-rh-precuneus_2 | frontal | parietal | .013 |
| 0,083891659 | .908 | ctx-rh-parsopercularis_1 | ctx-rh-superiorfrontal_4 | frontal | frontal | .013 |
| 0,081582238 | .884 | ctx-rh-medialorbitofrontal_2 | ctx-rh-middletemporal_2 | frontal | temporal | .011 |
| 0,082837078 | .879 | ctx-rh-parsopercularis_1 | ctx-rh-parstriangularis_1 | frontal | frontal | .012 |
| 0,080058768 | .845 | ctx-lh-inferiorparietal_2 | ctx-lh-parstriangularis_1 | parietal | frontal | .013 |
| 0,079840915 | .832 | ctx-rh-parsorbitalis_1 | ctx-rh-pericalcarine_1 | frontal | occipital | .013 |
| 0,079556848 | .83 | ctx-rh-fusiform_1 | ctx-rh-fusiform_2 | temporal | temporal | .021 |
| 0,078193703 | .797 | ctx-lh-precentral_4 | ctx-lh-superiortemporal_1 | frontal | temporal | .017 |
| -0,076284745 | .743 | ctx-lh-superiorfrontal_2 | ctx-rh-superiorfrontal_3 | frontal | frontal | .022 |
| 0,076237805 | .738 | ctx-rh-precuneus_2 | ctx-rh-superiorparietal_1 | parietal | parietal | .025 |
| 0,075036239 | .726 | ctx-lh-medialorbitofrontal_1 | ctx-lh-temporalpole_1 | frontal | temporal | .023 |
| 0,075241518 | .717 | ctx-lh-parsopercularis_1 | ctx-lh-insula_1 | frontal | insular | .023 |
| 0,074782485 | .702 | ctx-lh-paracentral_1 | ctx-lh-superiorparietal_1 | frontal | parietal | .033 |
| 0,074202685 | .699 | ctx-rh-entorhinal_1 | ctx-rh-parahippocampal_1 | temporal | temporal | .032 |
| -0,07382427 | .686 | ctx-rh-bankssts_1 | ctx-rh-middletemporal_2 | temporal | temporal | .03 |
| 0,073542434 | .679 | ctx-rh-parsopercularis_1 | ctx-rh-superiorfrontal_3 | frontal | frontal | .023 |
| -0,073839187 | .668 | ctx-lh-superiorfrontal_4 | ctx-rh-superiorfrontal_3 | frontal | frontal | .028 |
| -0,072255337 | .658 | ctx-lh-isthmuscingulate_1 | ctx-rh-superiorparietal_2 | parietal | parietal | .023 |
| 0,072870832 | .637 | ctx-rh-insula_1 | ctx-rh-insula_2 | insular | insular | .023 |
| -0,071988199 | .632 | ctx-lh-isthmuscingulate_1 | ctx-lh-precuneus_2 | parietal | parietal | .035 |
| 0,072116328 | .622 | ctx-rh-medialorbitofrontal_2 | ctx-rh-temporalpole_1 | frontal | temporal | .026 |
| -0,071988254 | .621 | ctx-lh-cuneus_1 | ctx-rh-cuneus_1 | occipital | occipital | .025 |
| -0,071024818 | .595 | ctx-lh-lateraloccipital_1 | ctx-rh-lateraloccipital_2 | occipital | occipital | .034 |
| -0,071026106 | .592 | ctx-lh-pericalcarine_1 | ctx-rh-lingual_1 | occipital | occipital | .032 |
| -0,070420964 | .586 | ctx-rh-lateraloccipital_2 | ctx-rh-parstriangularis_1 | occipital | frontal | .027 |
| -0,070213088 | .575 | ctx-lh-lingual_1 | ctx-lh-temporalpole_1 | occipital | temporal | .035 |
| -0,070010862 | .565 | ctx-rh-posteriorcingulate_1 | ctx-rh-precentral_3 | parietal | frontal | .024 |
| 0,069230478 | .551 | ctx-lh-superiorfrontal_1 | ctx-rh-frontalpole_1 | frontal | frontal | .042 |
| -0,068846213 | .543 | ctx-rh-isthmuscingulate_1 | ctx-rh-superiorparietal_3 | parietal | parietal | .041 |
| 0,068610765 | .527 | ctx-lh-fusiform_1 | ctx-lh-lateraloccipital_2 | temporal | occipital | .036 |
| -0,068938356 | .523 | ctx-rh-parsopercularis_1 | ctx-rh-supramarginal_1 | frontal | parietal | .045 |
| 0,068528179 | .521 | ctx-rh-precentral_3 | ctx-rh-superiorparietal_1 | frontal | parietal | .037 |
| 0,068613369 | .517 | ctx-lh-parstriangularis_1 | ctx-lh-rostralmiddlefrontal_3 | frontal | frontal | .04 |
| 0,068120685 | .517 | ctx-rh-postcentral_2 | ctx-rh-insula_1 | parietal | insular | .04 |
| 0,068136263 | .504 | ctx-lh-insula_1 | ctx-rh-precuneus_2 | insular | parietal | .035 |
| 0,0679331 | .502 | ctx-rh-paracentral_1 | ctx-rh-superiorparietal_1 | frontal | parietal | .046 |
| -0,067904244 | .499 | ctx-rh-superiorparietal_2 | ctx-rh-superiortemporal_2 | parietal | temporal | .034 |
| 0,068104186 | .497 | ctx-lh-precuneus_1 | ctx-rh-superiorparietal_2 | parietal | parietal | .036 |
| 0,066009132 | .443 | ctx-rh-entorhinal_1 | ctx-rh-precuneus_1 | temporal | parietal | .05 |
| 0,066589179 | .416 | ctx-lh-caudalanteriorcingulate_1 | ctx-lh-precuneus_1 | frontal | parietal | .05 |
| 0,065271499 | .399 | ctx-lh-lateraloccipital_1 | ctx-lh-medialorbitofrontal_1 | occipital | frontal | .043 |
| -0,065053318 | .357 | ctx-lh-parstriangularis_1 | ctx-lh-superiorfrontal_3 | frontal | frontal | .049 |
| 0,043087335 | .356 | ctx-lh-rostralmiddlefrontal_1 | ctx-rh-superiorfrontal_2 | frontal | frontal | .042 |
| 0,064964547 | .348 | ctx-rh-lingual_1 | ctx-rh-parsorbitalis_1 | occipital | frontal | .049 |
| -0,010364577 | .025 | ctx-rh-fusiform_1 | ctx-rh-rostralmiddlefrontal_2 | temporal | frontal | .048 |
| 0,00132722 | .014 | ctx-rh-lingual_1 | ctx-rh-temporalpole_1 | occipital | temporal | .003 |
| -0,000789199 | .006 | ctx-lh-lingual_2 | ctx-rh-precuneus_1 | occipital | parietal | .049 |
| 0,000483029 | .002 | ctx-lh-parstriangularis_1 | ctx-lh-precuneus_1 | frontal | parietal | .023 |
| 0,000149783 | .002 | ctx-rh-superiorfrontal_2 | ctx-rh-insula_1 | frontal | insular | .002 |

*Note.* Only edges with *p*<.05 are shown

# Supplementary Material 8: Detailed description of networks: FSozU

**Table 9**

*Negative network hubs FSozU*

| Node | Degree |
| --- | --- |
| ctx-rh-superiortemporal_1 | 5 |
| ctx-rh-superiorparietal_3 | 4 |
| ctx-rh-superiortemporal_2 | 4 |
| ctx-lh-parstriangularis_1 | 4 |
| ctx-lh-superiortemporal_1 | 3 |
| ctx-rh-precuneus_2 | 3 |
| ctx-lh-superiorparietal_3 | 3 |
| ctx-rh-superiorfrontal_2 | 3 |
| ctx-rh-transversetemporal_1 | 3 |

*Note.* The listed hubs are the top 15 % of nodes with the highest degree within the positive FSozU network. Abbreviations: lh = light hemisphere Abbreviations: lh = light hemisphere; rh = right hemisphere

**Table 10**

*Positive network hubs FSozU*

| Node | Degree |
| --- | --- |
| ctx-lh-postcentral_1 | 3 |
| ctx-rh-precuneus_2 | 3 |
| ctx-lh-precentral_1 | 3 |
| ctx-rh-superiorparietal_2 | 3 |
| ctx-rh-insula_1 | 2 |
| ctx-rh-superiorfrontal_2 | 2 |
| ctx-lh-fusiform_1 | 2 |

*Note.* The listed hubs are the top 15 % of nodes with the highest degree within the positive FSozU network. Abbreviations: lh = light hemisphere Abbreviations: lh = light hemisphere; rh = right hemisphere

**Figure 6**

*Associations of FSozU and the connectivity strength in positive and negative networks – original model*

*
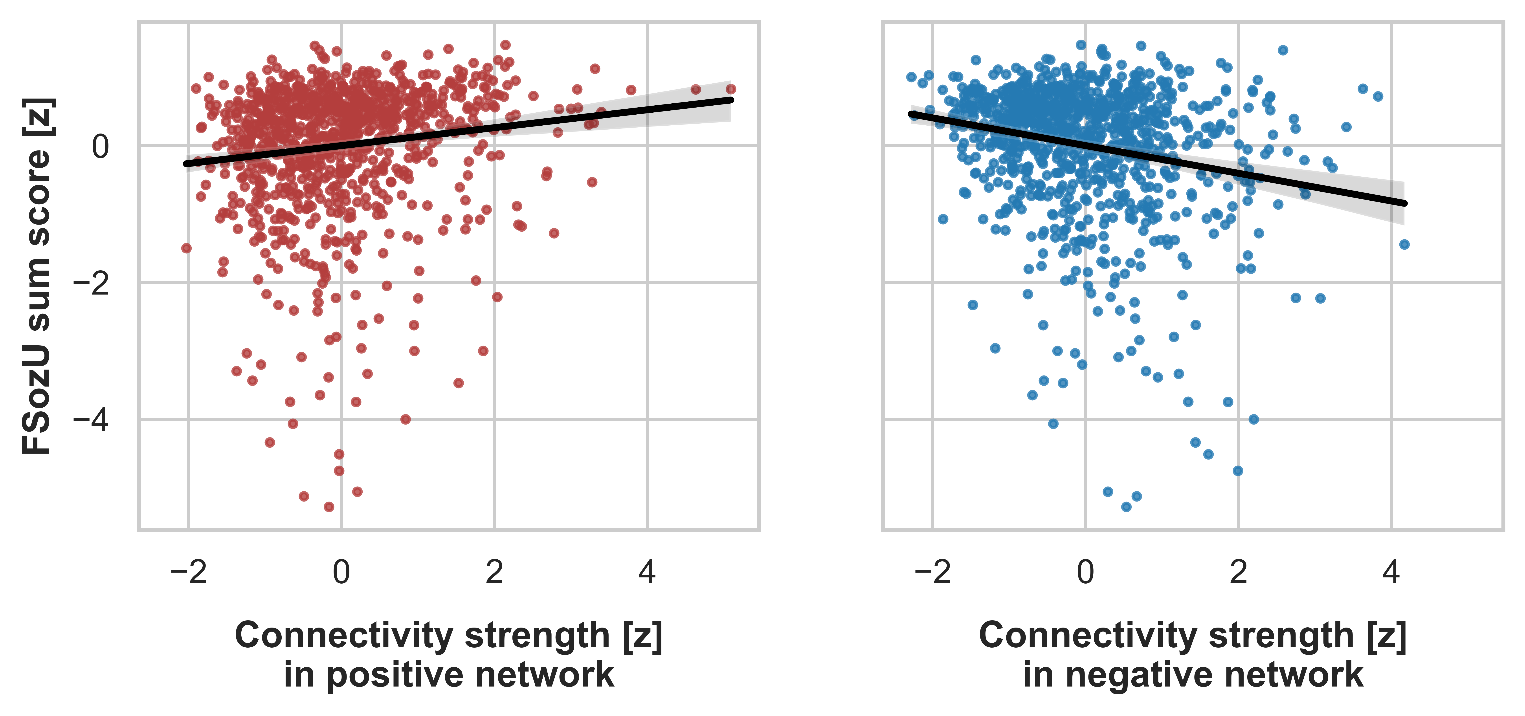
*

*Note.* The figure shows the association between the FSozU sum score and the connectivity strength (i.e., the total number of streamlines) of all edges that had a significant edge stability and were either positively (“positive network”) or negatively (“negative network”) associated to the FSozU. Note that FSozU values were corrected for influences of age, head motion, sex, total intracranial volume, site which can result in negative values.

**Figure 7**

*Figure 6 without multivariate outliers identified according to Mahalanobis distance*

**
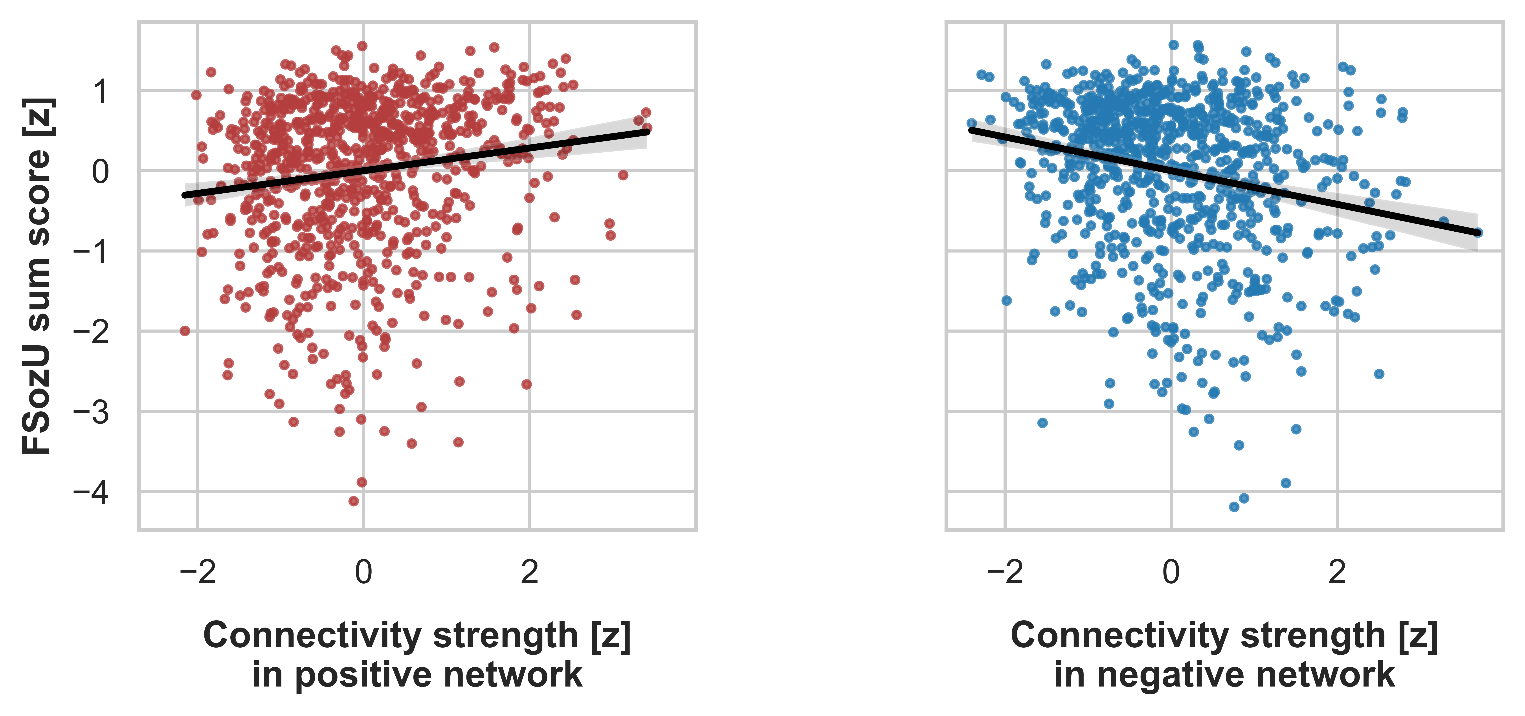
**

**Figure 8**

*Heatmaps of the regional distribution of brain regions*

*
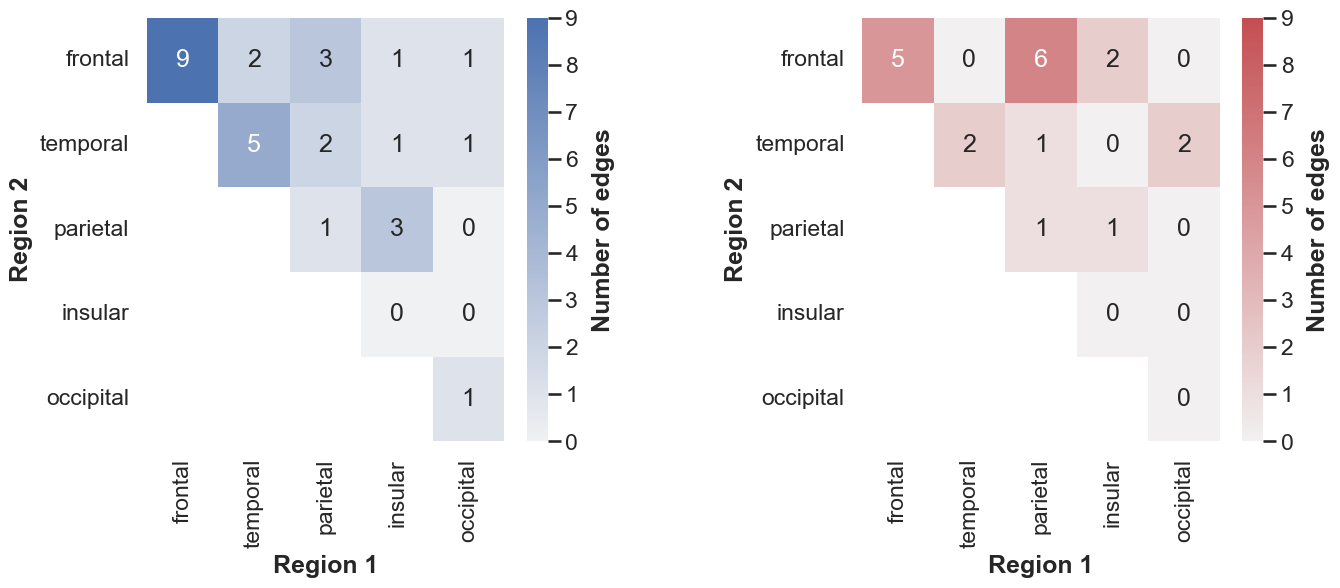
*

*Note.* The figure shows the number of network edges from the FSozU networks that connect frontal, temporal, parietal, and occipital brain regions. Red shows positive networks, blue negative networks.

**Table 11**

*Stability of significant edges associated with FSozU, and involved nodes*

| **Edge Correlation** | **Edge Stability (abs.)** | **Node 1** | **Node 2** | **Region 1** | **Region 2** | ***p*_EdgeStability_** |
| --- | --- | --- | --- | --- | --- | --- |
| -0.119599640591131 | 1 | lh-precentral_4 | lh-superiortemporal_1 | frontal | temporal | 0 |
| 0.0969553009230667 | .994 | lh-precentral_4 | lh-supramarginal_1 | frontal | parietal | .002 |
| 0.0933808625749032 | .993 | rh-precuneus_2 | rh-rostralanteriorcingulate_1 | parietal | frontal | .002 |
| 0.0958302105052517 | .987 | rh-lingual_2 | rh-superiorparietal_3 | occipital | parietal | .006 |
| 0.0936417454712958 | .983 | rh-parstriangularis_1 | rh-precuneus_2 | frontal | parietal | .004 |
| -0.0932977447831977 | .974 | rh-lateraloccipital_1 | rh-superiortemporal_1 | occipital | temporal | .007 |
| -0.0887757419890004 | .957 | rh-inferiortemporal_2 | rh-middletemporal_1 | temporal | temporal | .008 |
| -0.0889261587905366 | .956 | lh-bankssts_1 | lh-precentral_4 | temporal | frontal | .008 |
| 0.0872056797274098 | .945 | lh-precentral_1 | rh-superiorfrontal_3 | frontal | frontal | .01 |
| -0.0875216030558754 | .944 | rh-bankssts_1 | rh-superiortemporal_1 | temporal | temporal | .014 |
| -0.0884987383475501 | .943 | lh-superiortemporal_1 | lh-transversetemporal_1 | temporal | temporal | .01 |
| -0.085953487671565 | .932 | rh-parsorbitalis_1 | rh-superiortemporal_2 | frontal | temporal | .01 |
| 0.0864897490122514 | .919 | lh-rostralmiddlefrontal_3 | lh-superiorfrontal_2 | frontal | frontal | .009 |
| -0.0844911032265928 | .914 | lh-superiorparietal_1 | rh-precentral_3 | parietal | frontal | .01 |
| 0.0833419973033697 | .894 | lh-lateralorbitofrontal_2 | rh-medialorbitofrontal_1 | frontal | frontal | .007 |
| 0.0828229410753807 | .891 | rh-superiorparietal_2 | rh-superiortemporal_2 | parietal | temporal | .011 |
| 0.0817835867462444 | .881 | lh-rostralmiddlefrontal_3 | lh-insula_1 | frontal | insular | .01 |
| -0.0806002334811075 | .857 | rh-superiorparietal_3 | rh-superiortemporal_1 | parietal | temporal | .015 |
| 0.0807611061118965 | .853 | rh-middletemporal_1 | rh-supramarginal_1 | temporal | parietal | .023 |
| -0.0804492636329327 | .853 | rh-superiorparietal_3 | rh-insula_1 | parietal | insular | .013 |
| -0.0794771500438584 | .823 | rh-superiorparietal_3 | rh-transversetemporal_1 | parietal | temporal | .015 |
| -0.0784116992731888 | .811 | lh-inferiorparietal_1 | lh-superiortemporal_1 | parietal | temporal | .016 |
| -0.0775433989633081 | .781 | rh-superiorparietal_3 | rh-superiortemporal_2 | parietal | temporal | .019 |
| -0.0768631916402467 | .765 | lh-lateralorbitofrontal_1 | lh-parsorbitalis_1 | frontal | frontal | .024 |
| -0.0762853573086888 | .747 | rh-lateralorbitofrontal_1 | rh-lateralorbitofrontal_2 | frontal | frontal | .025 |
| 0.0753791300136871 | .738 | rh-parstriangularis_1 | rh-superiorparietal_2 | frontal | parietal | .024 |
| -0.0757048938135645 | .737 | lh-superiorparietal_3 | lh-superiortemporal_2 | parietal | temporal | .024 |
| -0.0754703313497156 | .732 | lh-superiorparietal_2 | lh-insula_2 | parietal | insular | .02 |
| 0.0744596012319589 | .706 | lh-postcentral_1 | lh-precentral_2 | parietal | frontal | .017 |
| 0.0740590093376446 | .691 | lh-posteriorcingulate_1 | lh-rostralanteriorcingulate_1 | parietal | frontal | .028 |
| -0.0733879268157855 | .679 | rh-lateralorbitofrontal_2 | rh-lingual_1 | frontal | occipital | .024 |
| -0.0734627885881498 | .672 | rh-cuneus_1 | rh-precuneus_1 | occipital | parietal | .017 |
| 0.0732037483407571 | .658 | lh-entorhinal_1 | lh-temporalpole_1 | temporal | temporal | .028 |
| -0.0723249540534672 | .648 | rh-precentral_1 | rh-superiorfrontal_4 | frontal | frontal | .02 |
| -0.072845253856005 | .647 | lh-fusiform_2 | lh-lateraloccipital_2 | temporal | occipital | .031 |
| 0.0686876520496204 | .644 | lh-postcentral_2 | lh-insula_2 | parietal | insular | .032 |
| -0.0727012187155908 | .643 | rh-precuneus_2 | rh-superiorfrontal_2 | parietal | frontal | .027 |
| 0.0721372302649169 | .642 | lh-caudalmiddlefrontal_1 | lh-precentral_1 | frontal | frontal | .034 |
| -0.0725233328827554 | .639 | lh-parstriangularis_1 | lh-rostralmiddlefrontal_3 | frontal | frontal | .036 |
| -0.0720714748611142 | .629 | lh-rostralmiddlefrontal_2 | rh-superiorfrontal_2 | frontal | frontal | .033 |
| -0.0711161439081786 | .619 | lh-rostralanteriorcingulate_1 | rh-medialorbitofrontal_1 | frontal | frontal | .028 |
| -0.0707317954521728 | .61 | lh-bankssts_1 | lh-postcentral_3 | temporal | parietal | .024 |
| -0.0544876207872826 | .61 | lh-rostralmiddlefrontal_1 | rh-superiorfrontal_2 | frontal | frontal | .003 |
| -0.0624038531066929 | .607 | lh-superiorfrontal_4 | rh-precuneus_2 | frontal | parietal | .021 |
| -0.0707629403778098 | .598 | lh-parstriangularis_1 | lh-superiorparietal_3 | frontal | parietal | .033 |
| 0.0712619945035822 | .595 | lh-superiorfrontal_4 | rh-superiorfrontal_2 | frontal | frontal | .035 |
| 0.0706697888475255 | .592 | rh-caudalanteriorcingulate_1 | rh-precuneus_2 | frontal | parietal | .037 |
| 0.0701948015816705 | .584 | lh-postcentral_1 | lh-precentral_3 | parietal | frontal | .042 |
| -0.070170731782055 | .572 | rh-transversetemporal_1 | rh-insula_1 | temporal | insular | .023 |
| -0.0703958172155707 | .57 | rh-parsopercularis_1 | rh-superiorfrontal_4 | frontal | frontal | .04 |
| -0.0704194497827649 | .566 | lh-superiorparietal_3 | lh-insula_1 | parietal | insular | .035 |
| -0.0695858804805998 | .553 | rh-lateraloccipital_1 | rh-lateraloccipital_3 | occipital | occipital | .03 |
| 0.0688372375682586 | .528 | lh-postcentral_1 | lh-precentral_1 | parietal | frontal | .046 |
| 0.0681513874390935 | .498 | lh-parahippocampal_1 | lh-pericalcarine_1 | temporal | occipital | .047 |
| 0.0669350245938052 | .496 | rh-parsorbitalis_1 | rh-supramarginal_2 | frontal | parietal | .046 |
| -0.0681709215062429 | .494 | rh-superiortemporal_1 | rh-transversetemporal_1 | temporal | temporal | .043 |
| -0.0676611546966502 | .492 | rh-inferiorparietal_2 | rh-precentral_1 | parietal | frontal | .043 |
| -0.0677342508828819 | .484 | rh-inferiorparietal_3 | rh-superiortemporal_1 | parietal | temporal | .044 |
| -0.0675556643039242 | .471 | lh-isthmuscingulate_1 | lh-precuneus_1 | parietal | parietal | .035 |
| -0.0672747351519324 | .467 | lh-inferiorparietal_2 | lh-parstriangularis_1 | parietal | frontal | .048 |
| -0.0662169349861555 | .449 | rh-bankssts_1 | rh-superiortemporal_2 | temporal | temporal | .043 |
| -0.0654126290574942 | .39 | lh-parstriangularis_1 | lh-precuneus_2 | frontal | parietal | .048 |
| -0.0650827877499278 | .375 | rh-cuneus_1 | rh-superiortemporal_2 | occipital | temporal | .049 |
| -0.0654356374307556 | .373 | lh-lateralorbitofrontal_1 | lh-lateralorbitofrontal_2 | frontal | frontal | .048 |
| 0.00702049626156046 | .077 | lh-caudalmiddlefrontal_1 | lh-supramarginal_2 | frontal | parietal | .01 |
| -0.00408916755882098 | .028 | lh-isthmuscingulate_1 | lh-temporalpole_1 | parietal | temporal | .045 |
| -0.00111927952591341 | .01 | lh-rostralmiddlefrontal_1 | lh-insula_2 | frontal | insular | .003 |
| -0.000841651437361231 | .009 | rh-lingual_1 | rh-temporalpole_1 | occipital | temporal | .01 |

*Note.* Only edges with *p*<.05 are shown

# Supplementary Material 9: Detailed description of overlapping edges

**Table 12**

*Edges with significant overlap in CTQ and FSozU networks*

| Node 1 | Node 2 | Index Edge | Effect CTQ | Effect FSozU |
| --- | --- | --- | --- | --- |
| lh-inferiorparietal_2 | lh-parstriangularis_1 | 2859 | 1 | -1 |
| lh-parstriangularis_1 | lh-rostralmiddlefrontal_3 | 4586 | 1 | -1 |
| lh-precentral_4 | lh-superiortemporal_1 | 5507 | 1 | -1 |
| rh-cuneus_1 | rh-precuneus_1 | 10663 | 1 | -1 |
| lh-rostralmiddlefrontal_1 | rh-superiorfrontal_2 | 11325 | 1 | -1 |
| rh-parsopercularis_1 | rh-superiorfrontal_4 | 11598 | 1 | -1 |
| rh-lingual_1 | rh-temporalpole_1 | 12616 | 1 | -1 |
| lh-postcentral_1 | lh-precentral_2 | 3676 | -1 | 1 |
| lh-entorhinal_1 | lh-temporalpole_1 | 6047 | -1 | 1 |
| lh-rostralmiddlefrontal_3 | lh-insula_1 | 6311 | -1 | 1 |
| rh-superiorparietal_2 | rh-superiortemporal_2 | 12188 | -1 | 1 |

*Note.* Edges which are positively correlated with CTQ and negatively correlated with FSozU are highlighted in black (*N* = 7), edges which are negatively correlated with CTQ and positively correlated with FSozU in green (*N* = 4).

# Supplementary Material 10: Evaluation of outliers

**Table 13**

*Comparing coefficients of the original model and models excluding outliers*

| Model | Estimate | *SE* | ***t*** | *P* > \|*t*\| | 97.5% CI | |
| --- | --- | --- | --- | --- | --- | --- |
|  |  |  |  |  | *LL* | *UL* |
| CTQ |  |  |  |  |  |  |
| Original model |  |  |  |  |  |  |
| positive | 0.2349 | 0.034 | 6.968 | <.001 | 0.169 | 0.301 |
| negative | -0.1397 | 0.033 | -4.177 | <.001 | -0.205 | -0.074 |
| Multivariate outliers (Mahalanobis distance) |  |  |  |  |  |  |
| positive | 0.2076 | 0.035 | 5.890 | <.001 | 0.138 | 0.277 |
| negative | -0.1445 | 0.034 | -4.206 | <.001 | -0.212 | -0.077 |
| FSozU |  |  |  |  |  |  |
| Original model |  |  |  |  |  |  |
| positive | 0.1445 | 0.034 | 4.272 | <.001 | 0.078 | 0.211 |
| negative | -0.2380 | 0.034 | -6.929 | <.001 | -0.305 | -0.171 |
| Multivariate outliers (Mahalanobis distance) |  |  |  |  |  |  |
| positive | 0.1539 | 0.035 | 4.443 | <.001 | 0.086 | 0.222 |
| negative | -0.2431 | 0.035 | -6.850 | <.001 | -0.313 | -0.173 |

*Note.* This table shows the respective model coefficients after exclusion of multivariate outliers using Mahalanobis distance; Abbreviations: CTQ=childhood trauma questionnaire; FSozU=social support questionnaire; *SE*=standard error; CI=confidence interval; *LL*=lower limit of the 97.5% CI; *UL*=upper limit of the 97.5 % CI

# Supplementary Material 11: Quadratic age as covariate

For the CTQ, CPM analysis revealed a positive and a negative network, that significantly predicted CTQ values of the participants (positive: *r*=.228, 95%-CI [.219; .237], *p*<.001, MAE=6.033, 95%-CI [5.979; 6.086], *p*<.001; negative: *r*=.240, 95%-CI [.230; .249], *p*<.001, MAE=5.973, 95%-CI [5.916; 6.029], *p*<.001). Stability analyses revealed that 62 edges (corresponding to 0.9% of the entire connectome) had a significant (i.e., higher than would be expected for random data) stability across the CV iterations.

For the FSozU, CPM analysis revealed a positive and a negative network, that significantly predicted FSozU values of the participants (positive: *r*=.166, 95%-CI [.156; .175], *p*<.001, MAE=0.386, 95%-CI [0.383; 0.389], *p*<.001; negative: *r*=.140, 95%-CI [.131; .149], *p*<.001, MAE=0.392, 95%-CI [0.389; 0.395], *p*<.001). Stability analyses revealed that 62 edges (corresponding to 0.9% of the entire connectome) had a significant (i.e., higher than would be expected for random data) stability across the CV iterations. Stability analyses revealed that 66 edges (corresponding to 1.0% of the entire connectome) had a significant (i.e., higher than would be expected for random data) stability across the CV iterations.

Focusing on edges with a significant (i.e., higher than would be expected for random data) stability across the CV iterations revealed that overlapping edges were again exclusively inversely correlated. To be precise, 11 edges with significant edge stability were predictive of CTQ and FSozU, with 7 of them being positively correlated with CTQ and negatively correlated with FSozU, and 4 of them being negatively correlated with CTQ and positively correlated with FSozU (Table 12).

**Table 14**

*Edges with significant overlap in CTQ and FSozU networks*

| Node 1 | Node 2 | Index Edge | Effect CTQ | Effect FSozU |
| --- | --- | --- | --- | --- |
| lh-inferiorparietal_2 | lh-parstriangularis_1 | 2859 | 1 | -1 |
| lh-parstriangularis_1 | lh-rostralmiddlefrontal_3 | 4586 | 1 | -1 |
| lh-precentral_4 | lh-superiortemporal_1 | 5507 | 1 | -1 |
| rh-cuneus_1 | rh-precuneus_1 | 10663 | 1 | -1 |
| lh-rostralmiddlefrontal_1 | rh-superiorfrontal_2 | 11325 | 1 | -1 |
| rh-parsopercularis_1 | rh-superiorfrontal_4 | 11598 | 1 | -1 |
| rh-lingual_1 | rh-temporalpole_1 | 12616 | 1 | -1 |
| lh-postcentral_1 | lh-precentral_2 | 3676 | -1 | 1 |
| lh-entorhinal_1 | lh-temporalpole_1 | 6047 | -1 | 1 |
| lh-rostralmiddlefrontal_3 | lh-insula_1 | 6311 | -1 | 1 |
| rh-superiorparietal_2 | rh-superiortemporal_2 | 12188 | -1 | 1 |

# Supplementary Material 12: Number of streamlines as covariate

For the CTQ, CPM analysis revealed a positive and a negative network, that significantly predicted CTQ values of the participants (positive: *r*=.217, 95%-CI [.207; .226], *p*<.001, MAE=6.033, 95%-CI [5.978; 6.088], *p*<.001; negative: *r*=.253, 95%-CI [.244; .262], *p*<.001, MAE=5.959, 95%-CI [5.903; 6.015], *p*<.001). Stability analyses revealed that 61 edges (corresponding to 0.9% of the entire connectome) had a significant (i.e., higher than would be expected for random data) stability across the CV iterations.

For the FSozU, CPM analysis revealed a positive and a negative network, that significantly predicted FSozU values of the participants (positive: *r*=.185, 95%-CI [.175; .195], *p*<.001, MAE=0.383, 95%-CI [0.380; 0.387], *p*<.001; negative: *r*=.119, 95%-CI [.109; .129], *p*<.001, MAE=0.393, 95%-CI [0.390; 0.396], *p*<.001). Stability analyses revealed that 66 edges (corresponding to 1.0% of the entire connectome) had a significant (i.e., higher than would be expected for random data) stability across the CV iterations.

Focusing on edges with a significant (i.e., higher than would be expected for random data) stability across the CV iterations revealed that overlapping edges were again exclusively inversely correlated. To be precise, 10 edges with significant edge stability were predictive of CTQ and FSozU, with 5 of them being positively correlated with CTQ and negatively correlated with FSozU, and 5 of them being negatively correlated with CTQ and positively correlated with FSozU (Table 13).

**Table 15**

*Edges with significant overlap in CTQ and FSozU networks*

| Node 1 | Node 2 | Index Edge | Effect CTQ | Effect FSozU |
| --- | --- | --- | --- | --- |
| lh-precentral_4 | lh-superiortemporal_1 | 5507 | 1 | -1 |
| rh-cuneus_1 | rh-precuneus_1 | 10663 | 1 | -1 |
| lh-rostralmiddlefrontal_1 | rh-superiorfrontal_2 | 11325 | 1 | -1 |
| rh-parsopercularis_1 | rh-superiorfrontal_4 | 11598 | 1 | -1 |
| rh-lingual_1 | rh-temporalpole_1 | 12616 | 1 | -1 |
| lh-postcentral_1 | lh-precentral_2 | 3676 | -1 | 1 |
| lh-entorhinal_1 | lh-temporalpole_1 | 6047 | -1 | 1 |
| lh-rostralmiddlefrontal_3 | lh-insula_1 | 6311 | -1 | 1 |
| lh-superiorfrontal_4 | rh-superiorfrontal_3 | 11445 | -1 | 1 |
| rh-superiorparietal_2 | rh-superiortemporal_2 | 12188 | -1 | 1 |

# Supplementary Material 13: Potential effects of sex

The CTQ sum score did not differ significantly between men and women (*M*_men_=32.55, *M*_women_=32.63, *t*(902)=-0.120, *p*=.905). For the FSozU, a significant difference emerged: (*M*_men_=4.39, *M*_women_ =4.57, *U*=11030.50, *Z*=4.614, *p*<.001).

To examine this potential influence of sex on the structural connectome in more detail, on top of adding sex as a covariate of no interest in all our CPM analyses, we investigated interaction effects of sex and the number of streamlines (NOS) in the positive and negative networks of the identified networks related to CTQ and FSozU, respectively. To this end, we used an ANCOVA with CTQ and FSozU as dependent variables, respectively, and sex, scanner site, age, TIV and head motion as covariates. No significant interaction effects were found between sex and NOS in the ANCOVAs:

**Table 16**

*Interaction effects of sex and NOS in positive and negative networks*

| Model |  | *F* | *p* |
| --- | --- | --- | --- |
| positive CTQ network |  |  |  |
| sex*NOS |  | 0.178 | .673 |
| negative CTQ network |  |  |  |
| sex*NOS |  | 0.019 | .890 |
| positive FSozU network |  |  |  |
| sex*NOS |  | 0.209 | .648 |
| negative FSozU network |  |  |  |
| sex*NOS |  | 0.177 | .674 |

*Note.* NOS=number of streamlines.

**Table 17**

*Comparison of model performances from models predicting CTQ and FSozU from connectome only vs. from connectome and the respective other variable*

|  | *r* | 95% CI | | *p* | *MAE* | 95% CI | | *p* |
| --- | --- | --- | --- | --- | --- | --- | --- | --- |
|  |  | *LL* | *UL* |  |  | *LL* | *UL* |  |
| CTQ |  |  |  |  |  |  |  |  |
| predicted from connectome only |  |  |  |  |  |  |  |  |
| positive | .224 | .215 | .233 | <.001 | 6.027 | 5.974 | 6.081 | <.001 |
| negative | .233 | .223 | .243 | <.001 | 5.968 | 5.911 | 6.025 | <.001 |
| predicted from connectome + FSozU |  |  |  |  |  |  |  |  |
| positive | .382 | .373 | .391 | <.001 | 5.569 | 5.516 | 5.621 | <.001 |
| negative | .395 | .386 | .403 | <.001 | 5.532 | 5.478 | 5.586 | <.001 |
| FSozU |  |  |  |  |  |  |  |  |
| predicted form connectome only |  |  |  |  |  |  |  |  |
| positive | .149 | .141 | .157 | <.001 | 0.386 | 0.383 | 0.389 | .003 |
| negative | .119 | .109 | .128 | .002 | 0.392 | 0.389 | 0.395 | .016 |
| predicted from connectome + CTQ |  |  |  |  |  |  |  |  |
| positive | .376 | .367 | .386 | <.001 | 0.354 | 0.351 | 0.357 | <.001 |
| negative | .361 | .352 | .370 | <.001 | 0.355 | 0.352 | 0.358 | <.001 |

*Note.* The table compares the predictive performance of the negative and positive networks associated with the CTQ and FSozU which were identified when excluding (Connectome only) or excluding (Connectome + FSozU / Connectome + CTQ) the respective other variable. *r*=correlation between true and predicted values, *LL*=lower limit of the 95% CI, *UL*=upper limit of the 95% confidence interval, *p*=*p*-value from permutation test, *MAE*=mean absolute error calculated for the true and predicted values.

# Supplementary Material 14: Robustness check

To ensure that, for example, the FSozU-connectome association was not driven by the FSozU's association with the CTQ, we repeated both predictive analyses while including the other measure as an additional covariate. As expected, the performance of the models predicting CTQ and FSozU increased compared to our original models (see Table 11). Nevertheless, significant likelihood ratio tests comparing models including and excluding the connectome as a predictor demonstrated that the connectome remained a significant predictor of both CTQ (positive network: LR = 25.47, *p* < .001, *R*²_connectome_ = 0.022, negative network: LR = 30.59, *p* < .001, *R*²_connectome_ = 0.026) and FSozU despite the additional correction (positive network: LR = 18.48, *p* < .001, *R*²_connectome_ = 0.016, negative network: LR = 40.36, *p* < .001, *R*²_connectome_ = 0.036).

# Supplementary Material 15: Cross-site check

At both sites, correlations were highly significantly negative and of similar size, even though slightly higher in Münster (Table 18).

However, since this led to a significant reduction of our training set, it is important to consider that this generally also leads to a reduction in performance of the model. We diminished the overall training data available for each model by 35% and 54%, respectively, (Marburg: *N*=530; Münster: *N*=374 as compared to approx. 814 subjects for all folds of the 10-fold cross-validation), which impacts the model's performance and predictive accuracy.

Even though the machine never had the opportunity to learn the scanner characteristics of the respective other site, surprisingly, predicting Münster from Marburg led to the same results. Vice versa, unfortunately, the prediction was not sufficiently accurate (Table 19). The correlation between true and predicted values was significant, however, not the MAE.

**Table 18**

*Bivariate Pearson’s* r *between FSozU and CTQ subscale scores*

| Variable | 1. | |
| --- | --- | --- |
|  | Marburg (*N*=530) | Münster  (*N*=374) |
| 1. Perceived social support (FSozU) | - | - |
| 2. CTQ_Sum score | -.345** | -.412** |

*Note.* ***p*<.001

**Table 19**

*Predicting Marburg from Münster and vice versa*

| Variable | Training | Test | Network | *r* | *p* | MAE | *p* |  |
| --- | --- | --- | --- | --- | --- | --- | --- | --- |
| CTQ | Marburg | Münster | positive | .209 | <.001 | 5.836 | <.001 |  |
|  |  |  | negative | .262 | <.001 | 5.795 | <.001 |  |
|  | Münster | Marburg | positive | .204 | <.001 | 7.121 | .389 |  |
|  |  |  | negative | .186 | <.001 | 6.662 | .197 |  |
| FSozU | Marburg | Münster | positive | .151 | <.001 | 0.348 | <.001 |  |
|  |  |  | negative | .119 | .004 | 0.345 | .006 |  |
|  | Münster | Marburg | positive | .129 | .001 | 0.679 | .907 |  |
|  |  |  | negative | .122 | .002 | 0.747 | .934 |  |

*Note.* CTQ=Childhood Trauma Questionnaire; MAE=mean absolute error; FSozU=”Fragebogen für soziale Unterstützung“/questionnaire for perceived social support

# Supplementary Material 16: Current perceived stress

We used some measures of current ‘perceived stress’ which can be regarded as a proxy of current stressful life events – the perceived stress scale (PSS) and the Life Events Questionnaire (LEQ).

The 14-item perceived stress scale (PSS) measures stress experienced in the last four weeks and might be related to current maltreatment-induced stress [26].

The LEQ asks participants about stressful negative and positive life events in the past six months [27]. One item asks whether the respondent has been the victim of a violent crime. In our sample, only 16 subjects (1.77%) reported having experienced one in the last six months. Unfortunately, this item neither provides information about the perpetrator’s relation to the participant, nor the frequency of the events.

The Trauma History Questionnaire (THQ) [28], also collected in the MACS, asks for experiences of potentially traumatic events in a yes/no format (such as crimes, general disasters, sexual and physical violence). For each event experienced, respondents also indicate the frequency of the event as well as their age at the time of the event. Specific information is additionally requested for most questions (e.g., if a weapon was involved). For the six sexual and physical trauma questions, the respondent is also asked whether the experience was repeated and if so, how often and at what age. We exploratively checked how many participants indicated current maltreatment, i.e., if any repeated sexual or physical assault happened to them in the current or past year of assessment. This only applied to one participant (aged 29) who indicated that she was sexually molested by other people including her partner, starting from age 14 - 20 times in total - until the time of assessment. She had a CTQ sum score of 67 which is > 2 SDs above the mean in our sample of 32.60 (SD=8.73). Other participants either did not indicate age of the event or reported that it was a singular event.

Since the PSS sum score and the LEQ negative event score correlated significantly with the CTQ sum score, we conducted a linear regression with the number of streamlines in the positive and negative CTQ network, respectively, as dependent variable, CTQ, age, sex, head motion, and PSS and the LEQ negative event score as independent variables. The PSS and LEQ did not have a significant influence on the number of streamlines in the respective networks.

**Table 20**

*Effects of LEQ and PSS on the number of streamlines in the positive CTQ network*

| Effect | Beta | T | *p* |
| --- | --- | --- | --- |
| Intercept |  | -.458 | .647 |
| Age | -.047 | -1.404 | .161 |
| Sex | -.074 | -1.842 | .066 |
| Head motion | .166 | 5.117 | **<.001** |
| TIV | .256 | 6.327 | **<.001** |
| PSS sum | .041 | 1.243 | .214 |
| CTQ sum | .199 | 5.838 | **<.001** |
| LEQ (negative events) | -.020 | -.612 | .541 |

*Note.* TIV=total intracranial volume; PSS=perceived stress scale; CTQ=childhood trauma questionnaire; LEQ=life events questionnaire

**Table 21**

*Effects of LEQ and PSS on the number of streamlines in the negative CTQ network*

| Effect | Beta | T | *p* |
| --- | --- | --- | --- |
| Intercept |  | .957 | .339 |
| Age | .003 | .100 | .920 |
| Sex | .049 | 1.184 | .237 |
| Head motion | -.076 | -2.265 | .024 |
| TIV | .284 | 6.855 | **<.001** |
| PSS | -.037 | -1.093 | .275 |
| CTQ | -.112 | -3.195 | **.001** |
| LEQ (negative events) | -.055 | -1.647 | .100 |

*Note.* TIV=total intracranial volume; PSS=perceived stress scale; CTQ=childhood trauma questionnaire; LEQ=life events questionnaire

# Supplementary Material 17: Supplementary Discussion on low levels of CM

The numbers of CM in our sample are relatively comparable to CM prevalence rates in Germany [29–31]. This might be due to the fact that our sample was younger on average as in the before mentioned studies, and physical neglect was more prevalent in Germany in the post-war period, without this being a symptom of physical neglect by the caregivers [32]. Higher maltreatment rates would have most likely been associated with a higher likelihood of mental disorder or depressive/anxious symptomatology [33, 34]. This would have in turn probably led to lower levels of social support, a) due to the negative correlation between these two measures [35] and b) due to social withdrawal as a common symptom or consequence of Major Depressive Disorder and Anxiety Disorders. This hypothesis is supported by the finding that healthy and depressed individuals are only slightly distinguishable at the neurobiological level, whereas childhood maltreatment and social support are the measures that best differentiate between these two groups [3]. It is important to note that the effects we found only apply to a subgroup of the general population which has not experienced severe CM.

Regarding the shared variance of CTQ and FSozU, a non-linear association between CTQ and FSozU is possible. With high levels of CM, it might be that the protective effect of social support starts to fade and other protective factors gain more importance. In our sample, there were *N*=269 with one scale above the respective cut-off (*r*(267)=-.241, *p*<.001; M_FsozU_=4.31), and *N*=108 with ≥ 2 scales above the respective cut-off (*r*(106)=-.086, *p*<.188; M_FsozU_ =4.16). This shows that indeed, examining the bivariate correlation between CTQ and FSozU in individuals with one or two subscales above an established cut-off [36] shows that the strength of association starts to decrease and eventually becomes non-significant. However, this might be due to the decreasing sample size and resulting loss of power. Future studies should examine our results in populations with severe CM.

# Supplementary references

1. Meinert S, Repple J, Nenadic I, Krug A, Jansen A, Grotegerd D, et al. Reduced fractional anisotropy in depressed patients due to childhood maltreatment rather than diagnosis. Neuropsychopharmacology. 2019;44:2065–2072.

2. Winter A, Thiel K, Meinert S, Lemke H, Waltemate L, Breuer F, et al. Familial risk for major depression: differential white matter alterations in healthy and depressed participants. Psychol Med. 2022:1–10.

3. Winter NR, Leenings R, Ernsting J, Sarink K, Fisch L, Emden D, et al. Quantifying Deviations of Brain Structure and Function in Major Depressive Disorder Across Neuroimaging Modalities. JAMA Psychiatry. 2022:1–11.

4. Kliem S, Mößle T, Rehbein F, Hellmann DF, Zenger M, Brähler E. A brief form of the Perceived Social Support Questionnaire (F-SozU) was developed, validated, and standardized. J Clin Epidemiol. 2015;68:551–562.

5. Lin M, Wolke D, Schneider S, Margraf J. Bullying History and Mental Health In University Students: The Mediator Roles of Social Support, Personal Resilience, and Self-Efficacy. Front Psychiatry. 2020;10.

6. Lin M, Hirschfeld G, Margraf J. Brief form of the Perceived Social Support Questionnaire (F-SozU K-6): Validation, norms, and cross-cultural measurement invariance in the USA, Germany, Russia, and China. Psychol Assess. 2019;31:609–621.

7. Chmitorz A, Neumann RJ, Kollmann B, Ahrens KF, Öhlschläger S, Goldbach N, et al. Longitudinal determination of resilience in humans to identify mechanisms of resilience to modern-life stressors: the longitudinal resilience assessment (LORA) study. Eur Arch Psychiatry Clin Neurosci. 2021;271:1035–1051.

8. Ahrens KF, Neumann RJ, Kollmann B, Brokelmann J, von Werthern NM, Malyshau A, et al. Impact of COVID-19 lockdown on mental health in Germany: longitudinal observation of different mental health trajectories and protective factors. Transl Psychiatry. 2021;11:1–10.

9. Struck N, Krug A, Feldmann M, Yuksel D, Stein F, Schmitt S, et al. Attachment and social support mediate the association between childhood maltreatment and depressive symptoms. J Affect Disord. 2020;273:310–317.

10. Flinkenflügel K, Meinert S, Thiel K, Winter A, Goltermann J, Strathausen L, et al. Negative Stressful Life Events and Social Support Are Associated With White Matter Integrity in Depressed Patients and Healthy Control Participants: A Diffusion Tensor Imaging Study. Biol Psychiatry. 2023. 6 April 2023. https://doi.org/10.1016/j.biopsych.2023.03.022.

11. Förster K, Danzer L, Redlich R, Opel N, Grotegerd D, Leehr EJ, et al. Social support and hippocampal volume are negatively associated in adults with previous experience of childhood maltreatment. J Psychiatry Neurosci. 2021;46.

12. Vogelbacher C, Möbius TWD, Sommer J, Schuster V, Dannlowski U, Kircher T, et al. The Marburg-Münster Affective Disorders Cohort Study (MACS): A quality assurance protocol for MR neuroimaging data. NeuroImage. 2018;172:450–460.

13. Andersson JLR, Skare S. A Model-Based Method for Retrospective Correction of Geometric Distortions in Diffusion-Weighted EPI. NeuroImage. 2002;16:177–199.

14. Chang LC, Jones DK, Pierpaoli C. RESTORE: Robust estimation of tensors by outlier rejection. Magn Reson Med. 2005;53:1088–1095.

15. Chang LC, Walker L, Pierpaoli C. Informed RESTORE: A method for robust estimation of diffusion tensor from low redundancy datasets in the presence of physiological noise artifacts. Magn Reson Med. 2012;68:1654–1663.

16. Hagmann P, Cammoun L, Gigandet X, Meuli R, Honey CJ, Van Wedeen J, et al. Mapping the Structural Core of Human Cerebral Cortex. PLOS Biol. 2008;6:e159.

17. Cammoun L, Gigandet X, Meskaldji D, Thiran JP, Sporns O, Do KQ, et al. Mapping the human connectome at multiple scales with diffusion spectrum MRI. J Neurosci Methods. 2012;203:386–397.

18. Desikan RS, Ségonne F, Fischl B, Quinn BT, Dickerson BC, Blacker D, et al. An automated labeling system for subdividing the human cerebral cortex on MRI scans into gyral based regions of interest. NeuroImage. 2006;31:968–980.

19. de Lange SC, Scholtens LH, van den Berg LH, Boks MP, Bozzali M, Cahn W, et al. Shared vulnerability for connectome alterations across psychiatric and neurological brain disorders. Nat Hum Behav 2019 39. 2019;3:988–998.

20. Repple J, Gruber M, Mauritz M, de Lange SC, Winter NR, Opel N, et al. Shared and Specific Patterns of Structural Brain Connectivity Across Affective and Psychotic Disorders. Biol Psychiatry. 2022. 21 June 2022. https://doi.org/10.1016/J.BIOPSYCH.2022.05.031.

21. Mori S, Van Zijl PCM. Fiber tracking: principles and strategies – a technical review. NMR Biomed. 2002;15:468–480.

22. Sarwar T, Ramamohanarao K, Zalesky A. Mapping connectomes with diffusion MRI: deterministic or probabilistic tractography? Magn Reson Med. 2019;81:1368–1384.

23. de Reus MA, van den Heuvel MP. Estimating false positives and negatives in brain networks. NeuroImage. 2013;70:402–409.

24. Zalesky A, Fornito A, Cocchi L, Gollo LL, van den Heuvel MP, Breakspear M. Connectome sensitivity or specificity: which is more important? NeuroImage. 2016;142:407–420.

25. Ponsoda V, Martínez K, Pineda-Pardo JA, Abad FJ, Olea J, Román FJ, et al. Structural brain connectivity and cognitive ability differences: A multivariate distance matrix regression analysis. Hum Brain Mapp. 2017;38:803–816.

26. Cohen S, Kamarck T, Mermelstein R. A Global Measure of Perceived Stress. J Health Soc Behav. 1983;24:385–396.

27. Norbeck JS. Modification of life event questionnaires for use with female respondents. Res Nurs Health. 1984;7:61–71.

28. Hooper LM, Stockton P, Krupnick JL, Green BL. Development, Use, and Psychometric Properties of the Trauma History Questionnaire. J Loss Trauma. 2011;16:258–283.

29. Häuser W, Schmutzer G, Brähler E, Glaesmer H. Maltreatment in Childhood and Adolescence: Results From a Survey of a Representative Sample of the German Population. Dtsch Ärztebl Int. 2011;108:287.

30. Witt A, Glaesmer H, Jud A, Plener PL, Brähler E, Brown RC, et al. Trends in child maltreatment in Germany: comparison of two representative population-based studies. Child Adolesc Psychiatry Ment Health. 2018;12:24.

31. Witt A, Brown RC, Plener PL, Brähler E, Fegert JM. Child maltreatment in Germany: prevalence rates in the general population. Child Adolesc Psychiatry Ment Health. 2017;11:47.

32. Klinitzke G, Romppel M, Häuser W, Brähler E, Glaesmer H. Die deutsche Version des Childhood Trauma Questionnaire (CTQ) – psychometrische Eigenschaften in einer bevölkerungsrepräsentativen Stichprobe. PPmP - Psychother · Psychosom · Med Psychol. 2012;62:47–51.

33. Nelson J, Klumparendt A, Doebler P, Ehring T. Childhood maltreatment and characteristics of adult depression: Meta-analysis. Br J Psychiatry. 2017;210:96–104.

34. Arango C, Dragioti E, Solmi M, Cortese S, Domschke K, Murray RM, et al. Risk and protective factors for mental disorders beyond genetics: an evidence-based atlas. World Psychiatry Off J World Psychiatr Assoc WPA. 2021;20:417–436.

35. Allen SF, Gilbody S, Atkin K, van der Feltz‐Cornelis CM. The associations among childhood trauma, loneliness, mental health symptoms, and indicators of social exclusion in adulthood: A UK Biobank study. Brain Behav. 2023;13:e2959.

36. Walker HE, Freud JS, Ellis RA, Fraine SM, Wilson LC. The Prevalence of Sexual Revictimization: A Meta-Analytic Review. Trauma Violence Abuse. 2019;20:67–80.
